# Polydopamine Nanotubes: Multifunctional Smart Nanotransducers for Cellular Activity Modulation

**DOI:** 10.1021/acsnano.6c05512

**Published:** 2026-08-07

**Authors:** Matteo Battaglini, Attilio Marino, Tommaso Curiale, Alessio Carmignani, Margherita Bernardeschi, Melis Emanet, Maria Cristina Ceccarelli, Federico Catalano, Filippo Drago, Sergio Marras, Giammarino Pugliese, Riccardo Carzino, Bruno Torre, Gianni Ciofani

**Affiliations:** † Smart Bio-Interfaces, 403538 Istituto Italiano di Tecnologia, Viale Rinaldo Piaggio 34, Pontedera 56025, Italy; ‡ Scuola Superiore Sant’Anna, The Biorobotics Institute, Viale Rinaldo Piaggio 34, Pontedera 56025, Italy; § Electron Microscopy Facility, Istituto Italiano di Tecnologia, Via Morego 30, Genova 16163, Italy; ∥ Nanochemistry, Istituto Italiano di Tecnologia, Via Morego 30, Genova 16163, Italy; ⊥ Materials Characterization Facility, Istituto Italiano di Tecnologia, Via Morego 30, Genova 16163, Italy; # Advanced Materials and Life Science Division, Istituto Nazionale di Ricerca Metrologica INRiM, Strada delle Cacce 91, Torino 10135, Italy

**Keywords:** polydopamine nanotubes, remote cellular stimulation, piezoelectric nanoparticles, NIR-responsive nanoparticles, antioxidant nanoparticles, dopamine release

## Abstract

Polydopamine nanotubes (PDA-NTs) represent a recently developed and scarcely explored class of nanostructures with exceptional multifunctionality. Synthesized through a ZnO-templated Stöber-like process followed by template removal, PDA-NTs exhibit a combination of piezoelectric, photothermal, and antioxidant properties, a collection of features never observed together in a single organic nanoparticle. This work provides the demonstration of electromechanical conversion in polydopamine structures, with PDA-NTs showing a robust piezoelectric coefficient (*d*_33_ = 15.0 ± 0.7 pC/N), a property completely absent in spherical PDA nanoparticles. PDA-NTs are efficiently internalized by cells and display excellent biocompatibility; upon remote stimulation, they can modulate intracellular calcium levels via ultrasound-driven activation or near-infrared (NIR) photothermal heating. In addition, their strong antioxidant capacity mitigates oxidative stress, while controlled dopamine release (both passive and ultrasound-triggered) induces functional effects in human neural stem cells. This unique combination of mechanical and NIR responsiveness, antioxidant activity, and neuromodulatory drug release within a single organic nanostructure marks a significant breakthrough, allowing PDA-NTs to emerge as a unique class of “smart” multifunctional nanomaterials, enabling remote cellular control and offering exciting opportunities for regenerative medicine, neurostimulation, and advanced bioelectronics.

## Graphical abstract

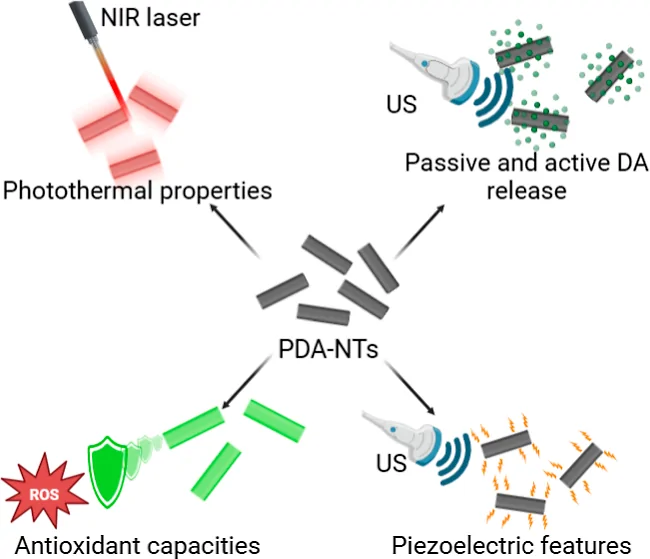

Polydopamine (PDA), a bioinspired polymer derived from the oxidative polymerization of dopamine (DA), is a highly versatile material in nanomedicine and bioengineering, with excellent biocompatibility, obtained through mild synthesis conditions, and multifunctional surface chemistry.^1–4^ Inspired by the adhesive proteins secreted by mussels, PDA strongly adheres to a wide range of substrates and can be exploited as an intermediate linker for further functionalization.^3^ Moreover, PDA nanostructures serve as efficient photothermal conversion agents, converting irradiation at specific wavelengths (e.g., near-infrared -NIR-) into heat for therapeutic and technological applications.^5,6^ These features have led to the exploitation of PDA in coatings, drug delivery, photothermal therapy, biosensing, and tissue scaffolds.^7–12^ Among PDA most known features, we can find its redox activity mediated by the presence of catechol groups, which grant antioxidant properties and chemical versatility for conjugating bioactive molecules or therapeutic agents.^2,13–15^

To date, most of the research involving PDA in biomedical applications has focused on its use in the form of spherical nanoparticles (PDA-NPs), which are relatively easy to synthesize and offer a high surface area for interaction with biological environments. PDA-NPs have shown promising results in delivering drugs, scavenging reactive oxygen species, and converting light into localized heat for photothermal applications.^1,8,11,14–19^

Nanostructure morphology is a critical design parameter that can dramatically influence physicochemical behavior, cellular interactions, and functional performance.^20^ In this regard, nanotubular morphologies have attracted increasing interest due to their anisotropic geometry, hollow cores, and increased surface-to-volume ratios.^21^ Despite the potential advantages of anisotropic shapes, polydopamine nanotubes (PDA-NTs) remain drastically underexplored in the scientific community.^21,22^ Only a handful of studies have described the synthesis of PDA-NTs using templating methods based on ZnO nanowires, curcumin, or clay nanotubes.^23–25^ These works primarily focused on catalysis, chemical sensing, or pH-dependent drug release. To our knowledge, no prior studies have investigated the behavior of PDA-NTs in cellular or biological systems, nor have any reports examined their electromechanical properties. This represents a major gap in the field, particularly given the well-established importance of geometry-dependent behavior in nanoscale systems.

Here, we address this gap by presenting the first comprehensive biological and functional characterization of PDA-NTs, revealing a rich set of emergent properties uniquely enabled by their tubular morphology. In contrast to spherical PDA-NPs, PDA-NTs exhibit robust piezoelectricity, a rare and highly valuable feature in organic nanomaterials. Our results demonstrate a significant piezoresponse (*d*_33_ = 15.0 ± 0.7 pC/N) in PDA-NTs, which is absent in PDA-NPs, highlighting that piezoelectricity is not an intrinsic property of PDA, but rather a shape-induced emergent functionality. In addition to their piezoelectricity, we demonstrate that PDA-NTs, similarly to PDA-NPs, exhibit strong photothermal conversion efficiency under NIR irradiation and high antioxidant capacity, making them suitable for use as cytoprotective agents against oxidative stress.

For the first time, we explore the biocompatibility, internalization, and functional activity of PDA-NTs in cellular models, including their ability to modulate intracellular calcium signaling through both ultrasound (US)-driven piezoelectric stimulation and light-induced photothermal activation. Furthermore, we demonstrate that PDA-NTs can release DA both passively and in response to US, and that the released DA maintains its activity, as evidenced by the response of human neural stem cells.

Together, these findings establish PDA-NTs as a unique class of multifunctional nanostructures, capable of integrating mechanical-to-electrical transduction, photothermal responsiveness, antioxidant protection, and neuromodulatory drug release within a single, biocompatible and biodegradable platform. The demonstration, for the first time in the literature, that PDA-NTs can actively interface with biological systems marks a critical advance in the design of innovative, organic nanomaterials. These results open new perspectives for PDA-based nanostructures applications in regenerative medicine, neural interfacing, oncology, and responsive bioelectronics, where geometry-dependent properties can be tailored and exploited for precise, multimodal therapeutic strategies.

## Results

### Polydopamine Nanotubes Synthesis and Characterization

ZnO nanowires, polydopamine-coated ZnO nanowires (PDA-ZnO), PDA-NTs, and PDA-NPs were characterized using scanning electron microscopy (SEM) and transmission electron microscopy (TEM), as shown in Figure 1a. The analyses highlighted the distinct structural characteristics and morphology of the PDA-NTs compared to the other three nanostructures. TEM observations confirmed the presence of ZnO nanowires embedded in the PDA-ZnO composite (indicated by arrows in Figure 1b, left panel), no longer detectable after the etching process (Figure 1b, right panel), as also confirmed by the energy-dispersive spectroscopy (EDS) results (Figure 1c). PDA-NTs were estimated to be 1090 ± 580 nm in length and 140 ± 40 nm in diameter; dynamic light scattering (DLS) analysis (Figure S1) showed a hydrodynamic size of 1103 ± 36 nm and a ζ-potential of −16.5 ± 1.3 mV.

**Figure 1:**
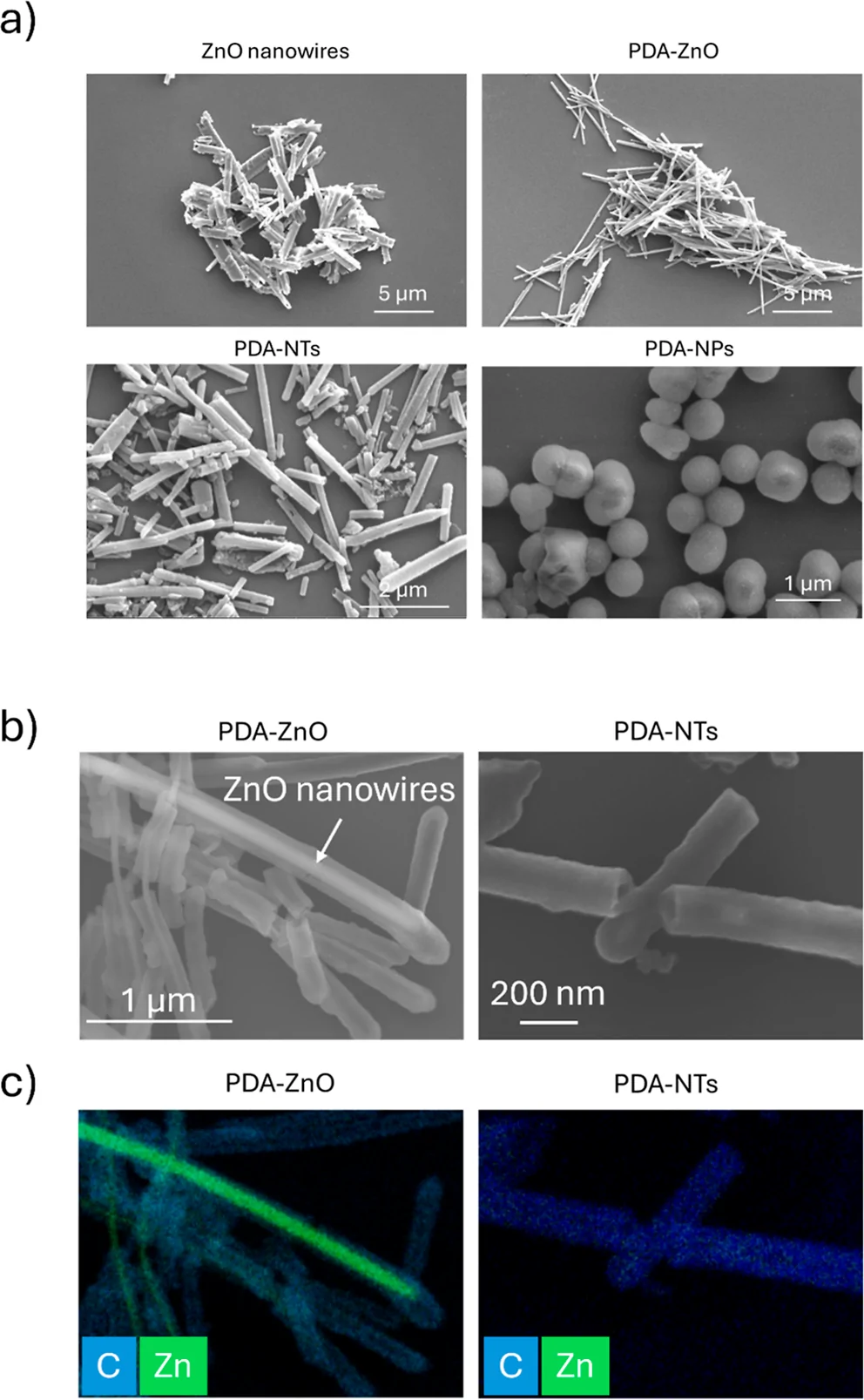
(a) Representative SEM images of ZnO nanowires, PDA-ZnO, PDA-NTs and PDA-NPs; (b) representative TEM images of PDA-ZnO and PDA-NTs; (c) EDS analysis for PDA-ZnO and PDA-NTs showing the distribution of carbon (in blue) and zinc (in green).

A comprehensive set of characterization analyses was conducted to reliably confirm the removal of the ZnO template and to chemically characterize PDA-NTs. Thermogravimetric analysis (TGA) results confirmed both the successful elimination of the ZnO core and the distinct thermal behavior of the obtained nanostructures (Figure 2a). The derivative weight curves revealed a sharp degradation peak for pristine ZnO nanowires at 256.2 °C. For the PDA-ZnO composite, this peak slightly shifted to 266.7 °C. Conversely, PDA-NTs showed no distinct peak in this region, instead displaying a broad and gradual weight loss profile. Inductively coupled plasma (ICP) analysis quantitatively assessed the zinc content in PDA-ZnO and PDA-NTs: PDA-ZnO showed a Zn content of 76.0 ± 0.1%, in contrast to the 1.5 ± 0.1% of PDA-NTs.

**Figure 2:**
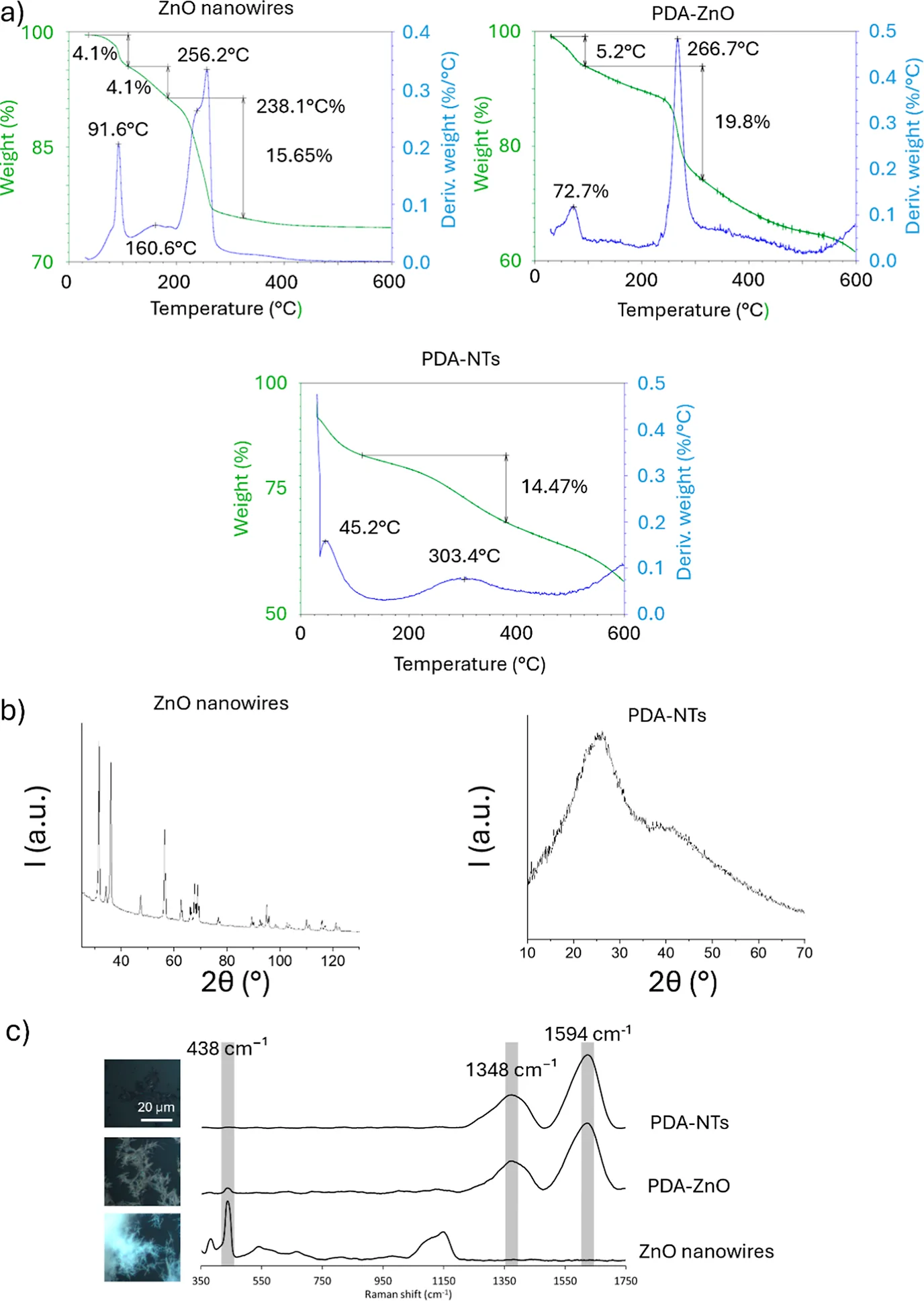
(a) TGA analysis of ZnO nanowires, PDA-ZnO, and PDA-NTs; (b) XRD analysis of ZnO nanowires and PDA-NTs. (c) Representative bright field images of the scanned areas (on the left) and Raman spectra of ZnO nanowires, PDA-ZnO, and PDA-NTs.

X-ray diffraction (XRD) analysis provided additional confirmation of ZnO elimination and PDA-NT formation (Figure 2b). The pristine ZnO nanowires exhibited sharp, intense reflections characteristic of the wurtzite crystalline phase (Figure 2b, left).^26^ Following NH_4_Cl treatment, these peaks are not present anymore, being substituted by a broad, low-intensity peak at *2*θ ∼25°, associated with the amorphous structure of PDA (Figure 2b, right).^27^

Finally, Raman spectroscopy (Figure 2c) was carried out; a distinct peak at 438 cm^–1^, present in ZnO and PDA–ZnO samples but absent in PDA-NTs, confirmed the removal of ZnO. Conversely, two peaks at 1348 cm^–1^ and 1594 cm^–1^ are present in both PDA-NTs and PDA-ZnO, but not in ZnO nanowires.^28,29^

SEM and EDS analyses confirmed the progressive removal of the ZnO template during the etching process (Figure S2). While ZnO nanowires and PDA-ZnO samples displayed a strong Zn signal, PDA-NTs showed a marked reduction in Zn content with increasing etching time, becoming undetectable after 60 min. At the same time, SEM images revealed that the nanotubular morphology was preserved throughout the etching process, supporting the successful formation of hollow PDA nanotubes. X-ray photoelectron spectroscopy (XPS) analysis further confirmed the progressive removal of the ZnO template during PDA-NT formation (Figure S3). The Zn 2p_3/2_ peak showed a gradual decrease with increasing etching time, indicating the reduction of residual Zn species after NH_4_Cl treatment. Consistently, elemental composition analysis revealed a marked decrease in the Zn atomic percentage, along with the predominance of C, O, and N signals associated with the polydopamine structure, supporting the successful formation of PDA nanotubes.

### Piezoelectric Properties

The surface features and piezoelectric properties of ZnO nanowires, PDA-ZnO, PDA-NTs, and PDA-NPs (the latter serving as internal control), were characterized by both Atomic force microscopy (AFM) and piezoelectric force microscopy (PFM). AFM confirmed the spherical shape of PDA-NPs (Figure 3a), and no significant piezoelectric response was observed by PFM (Figure 3b). The tubular shape of PDA-NTs was confirmed by AFM (Figure 3c), and in this case, a *d*_33_ of 15.0 ± 0.7 pC/N was observed through PFM analysis (Figure 3d).

**Figure 3:**
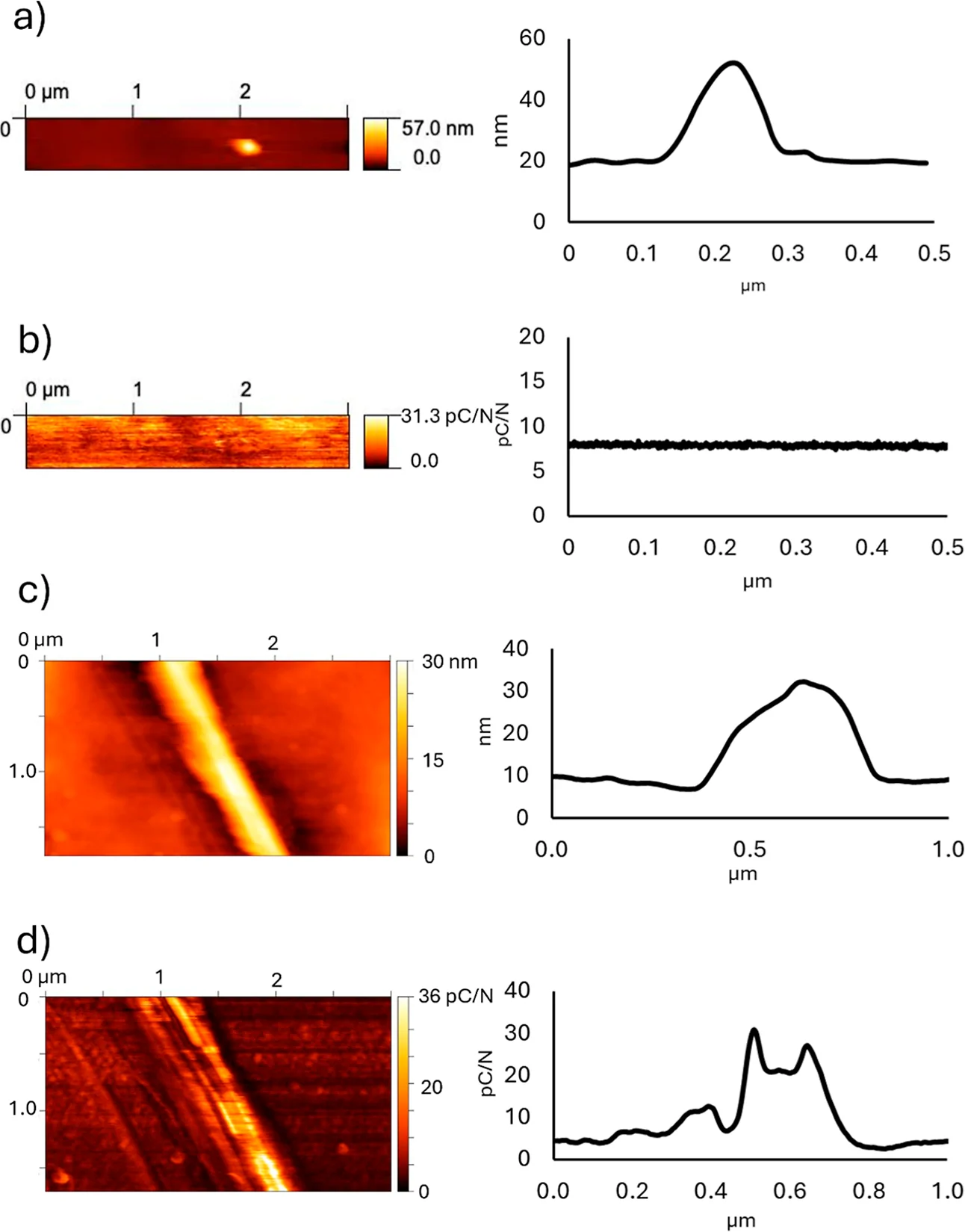
AFM (a, c) and PFM (b, d) characterization of PDA-NPs (a, b) and PDA-NTs (c, d). On the left representative images, on the right quantitative analysis.

AFM and PFM analyses performed over larger scan areas confirmed the homogeneous distribution of PDA-NTs and the preservation of their electromechanical activity across the sample surface (Figure S4). The AFM topography images showed a widespread nanotubular morphology, while the corresponding PFM *d*_33_ mapping revealed a consistent piezoelectric response associated with the nanotubes throughout the analyzed area. The tubular shape of ZnO and PDA-ZnO was also confirmed by AFM, and as expected, a significant piezoelectric response was measured through PFM. AFM analysis of ZnO is shown in Figure S5a, with an average *d*_33_ of 6.2 ± 0.7 pC/N (PFM, Figure S5b), while AFM analysis of PDA-ZnO is shown in Figure S5c, with an average *d*_33_ of 6.6 ± 1.5 pC/N (PFM, Figure S5d).

### Biocompatibility and Cellular Interaction

PDA-NT treatment did not significantly affect the viability of U87 MG cells at concentrations up to 100 μg/mL (Figure S6), with no detectable changes in the percentage of viable and necrotic cells (*p* > 0.05).

Figure S7 shows a remarkable PDA-NT uptake by U87 MG cells following 24 and 72 h of exposure, with partial colocalization observed within lysosomes at both time points (Pearson’s correlation coefficients of 0.26 ± 0.05 and 0.28 ± 0.08, respectively). We moreover investigated the internalization pathway of PDA-NTs using the water-soluble dye Cascade Blue as a marker of fluid-phase uptake. The observed colocalization between PDA-NTs and Cascade Blue (Figure S8), with a Pearson’s correlation coefficient of 0.20 ± 0.08 after 72 h of incubation, suggests that pinocytosis contributes to their cellular internalization. The intracellular localization of PDA-NTs is further illustrated by the 3D rendering shown in Figure S9. PDA-NTs were also associated with the cell membrane, where they appeared as tubular, electron-dense structures clearly identifiable by SEM imaging (Figure S10).

### Antioxidant Protective Effects of Polydopamine Nanotubes

The antioxidant activity of PDA-NTs and PDA-NPs was assessed using a Total Antioxidant Capacity Assay Kit (Figure S11). Both nanostructures exhibited comparable antioxidant capacities, with 10 and 50 μg/mL of PDA-NTs showing antioxidant features equivalent to Trolox 103.7 ± 6.0 μM and 253.9 ± 4.5 μM, respectively; 10 μg/mL PDA-NPs were instead equivalent to Trolox 96.4 ± 3.2 μM, while 50 μg/mL to 230.0 ± 8.0 μM.

The protective effects of PDA-NTs were evaluated in U87 MG cells exposed to a pro-oxidative stimulus (*tert*-butyl hydroperoxide, TBH). As shown in Figure 4a, treatment with TBH significantly increased cytosolic reactive oxygen species (ROS) levels (*p* < 0.001), with the percentage of ROS-positive cells rising from 12.9 ± 0.4% in control to 20.9 ± 0.5% and 26.6 ± **2.2%** after exposure to TBH 2.5 and 5.0 mM, respectively. PDA-NTs significantly reduced cytosolic ROS (*p* < 0.001), lowering the ROS-positive cells to 3.2 ± 0.2% in control conditions and to 7.4 ± 0.4% and 14.3 ± 0.6% in the presence of TBH 2.5 and 5.0 mM, respectively.

**Figure 4:**
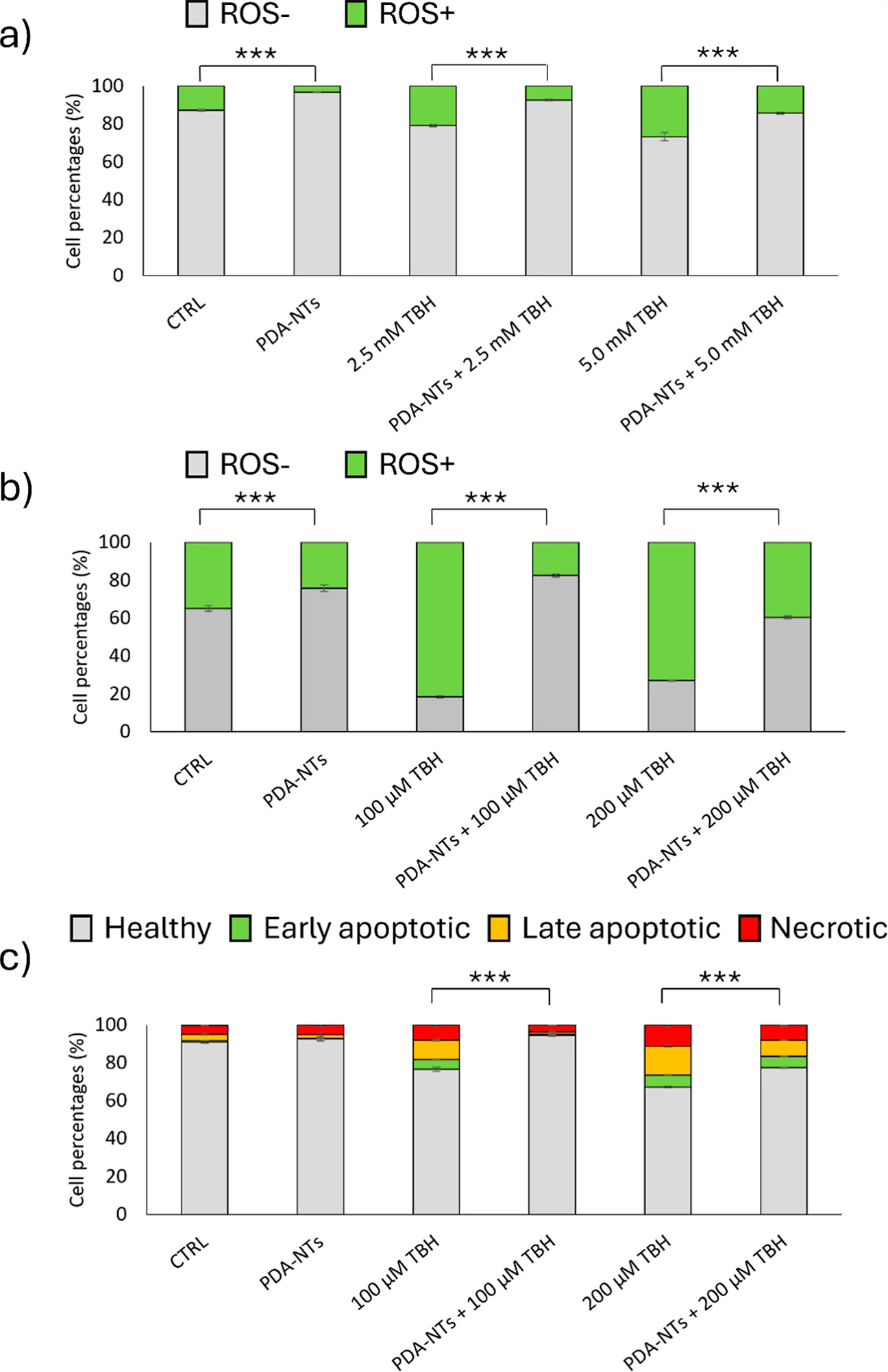
Flow cytometry analysis showing, for each experimental condition in U87 MG cells: (a) the relative levels of intracellular ROS, (b) the relative levels of mitochondrial ROS, and (c) the fractions of healthy, apoptotic, and necrotic cells (***p < 0.001).

A similar trend was observed for mitochondrial ROS (Figure 4b). TBH treatment strongly increased (*p* < 0.001) the number of cells positive for mitochondrial ROS, from 34.8 ± 1.4% in control to 81.5 ± 0.5% and 72.9 ± 0.3% in the presence of TBH 100 and 200 μM, respectively. PDA-NTs significantly reduced also mitochondrial ROS levels (*p* < 0.001), with ROS-positive cells decreasing to 24.1 ± 1.8% in control conditions, and to 17.5 ± 0.8% and 39.7 ± 0.8% in the presence of TBH 100 and 200 μM, respectively.

Eventually, TBH exposure significantly decreased cell viability (Figure 4c, *p* < 0.001), with the percentage of healthy cells decreasing from 90.9 ± 0.6% in control to 76.6 ± 1.1% and 67.3 ± 0.4% after treatment with TBH 100 and 200 μM, respectively. Notably, this effect was partially counteracted by PDA-NTs, which maintained cell viability at 94.4 ± 0.3% in the presence of TBH 100 μM and at 77.4 ± 0.4% in the presence of TBH 200 μM (*p* < 0.001).

Representative flow cytometry histograms for all these assays are reported in Figures S12–S14.

### Modulation of Cellular Activities through NIR and US Stimulation

The photothermal conversion properties of PDA-NTs were evaluated using NIR laser irradiation combined with thermal camera imaging (Figure 5a, representative thermo-images, and Figure 5b, quantitative evaluation). PDA-NTs exhibited efficient NIR-mediated photothermal conversion, producing a concentration-dependent temperature increase ranging from 6.0 ± 0.3 °C for 25 μg/mL to 17.0 ± 1.7 °C for 500 μg/mL. Notably, PDA-NTs outperformed PDA-NPs in terms of photothermal conversion properties, reaching a higher temperature increment (6.0 ± 0.3 °C for 25 μg/mL, 17.0 ± 1.7 °C for 500 μg/mL) compared to the spherical counterpart (3.1 ± 0.3 °C for 25 μg/mL, 15.4 ± 1.9 °C at 500 μg/mL).

**Figure 5:**
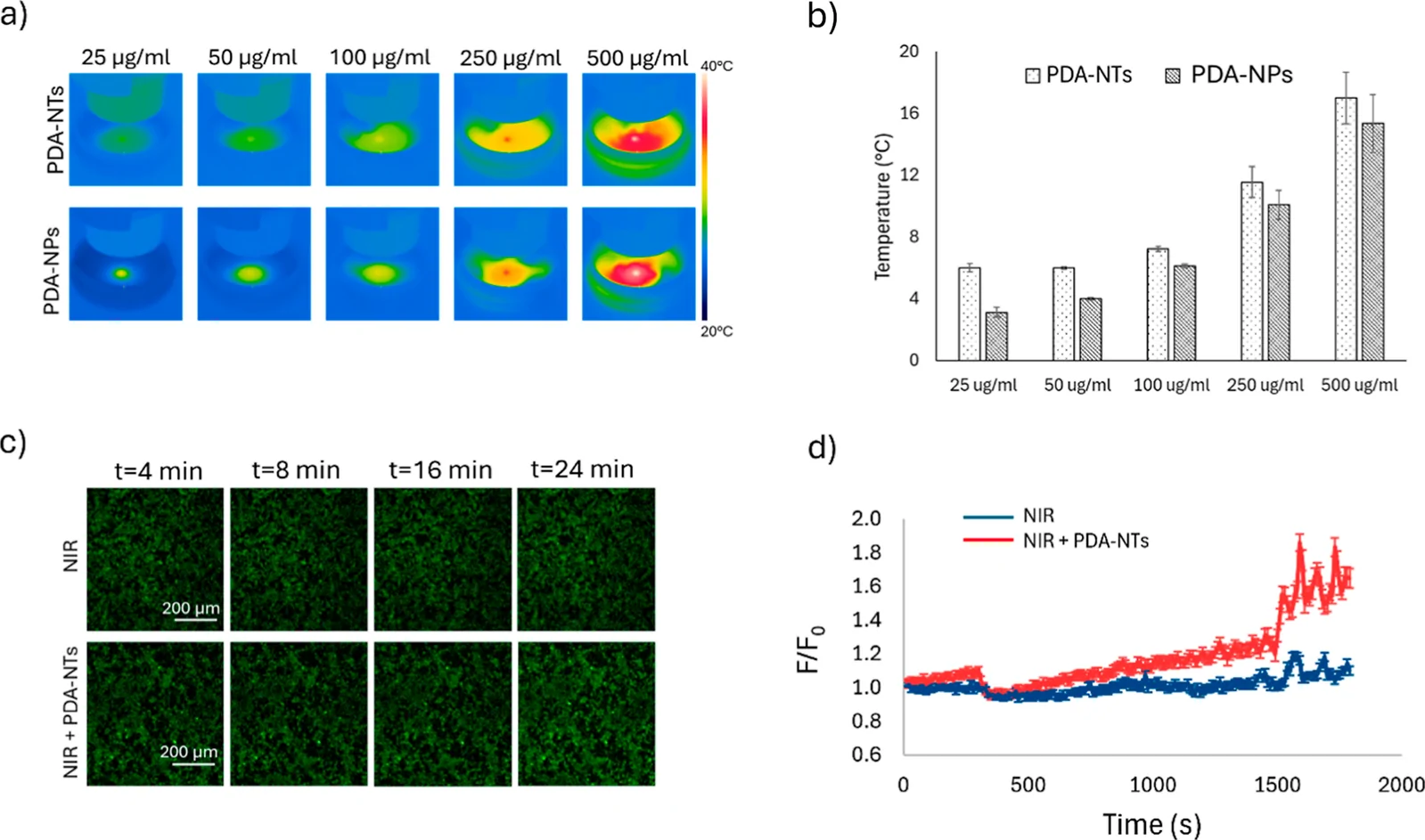
(a) Representative thermo-images of aqueous PDA-NT and PDA-NP dispersions of different concentrations under 808 nm NIR laser irradiation. (b) Quantitative analysis of the maximum temperature change (Δ*T*) after irradiation, for each PDA-NT and PDA-NP concentration. (c) Representative confocal images during calcium imaging of U87 MG cells under NIR irradiation, with and without PDA-NTs, and (d) quantitative analysis.

Moreover, as shown in Figure S15, PDA-NTs effectively increased the intracellular temperature of U87 MG cells upon NIR laser irradiation. Cells treated with PDA-NTs exhibited a temperature rise of 7.8 ± 3.4 °C, while no significant changes were observed under control conditions with NIR irradiation alone. This property was subsequently harnessed to modulate intracellular calcium levels in U87 MG cells. As shown in Figure 5c (representative confocal images) and Figure 5d (quantitative evaluation), plain NIR irradiation did not alter intracellular calcium concentrations. Conversely, the combined treatment with PDA-NTs and NIR resulted in a ∼50% increase in intracellular calcium levels over the irradiation period.

The piezoelectric properties of PDA-NTs were also exploited, as proof of concept, for their ability to remotely regulate intracellular calcium levels in cells upon US stimulation. Treatment with plain US (Figure 6a, Video S1) or with US in combination of nonpiezoelectric PDA-NPs (Figure 6b) did not produce any significant increase in intracellular calcium levels; conversely, when US are applied in the presence of piezoelectric PDA-NTs transient elevations in intracellular calcium are triggered (Figure 6c, Video S2). Moreover, as shown in Figure S16, no significant increase in intracellular calcium levels was detected in cells treated with PDA-NTs and exposed to US in the presence of CdCl_2_ or in Ca^2+^-free PBS, suggesting that the observed calcium response depends on extracellular calcium influx.

**Figure 6:**
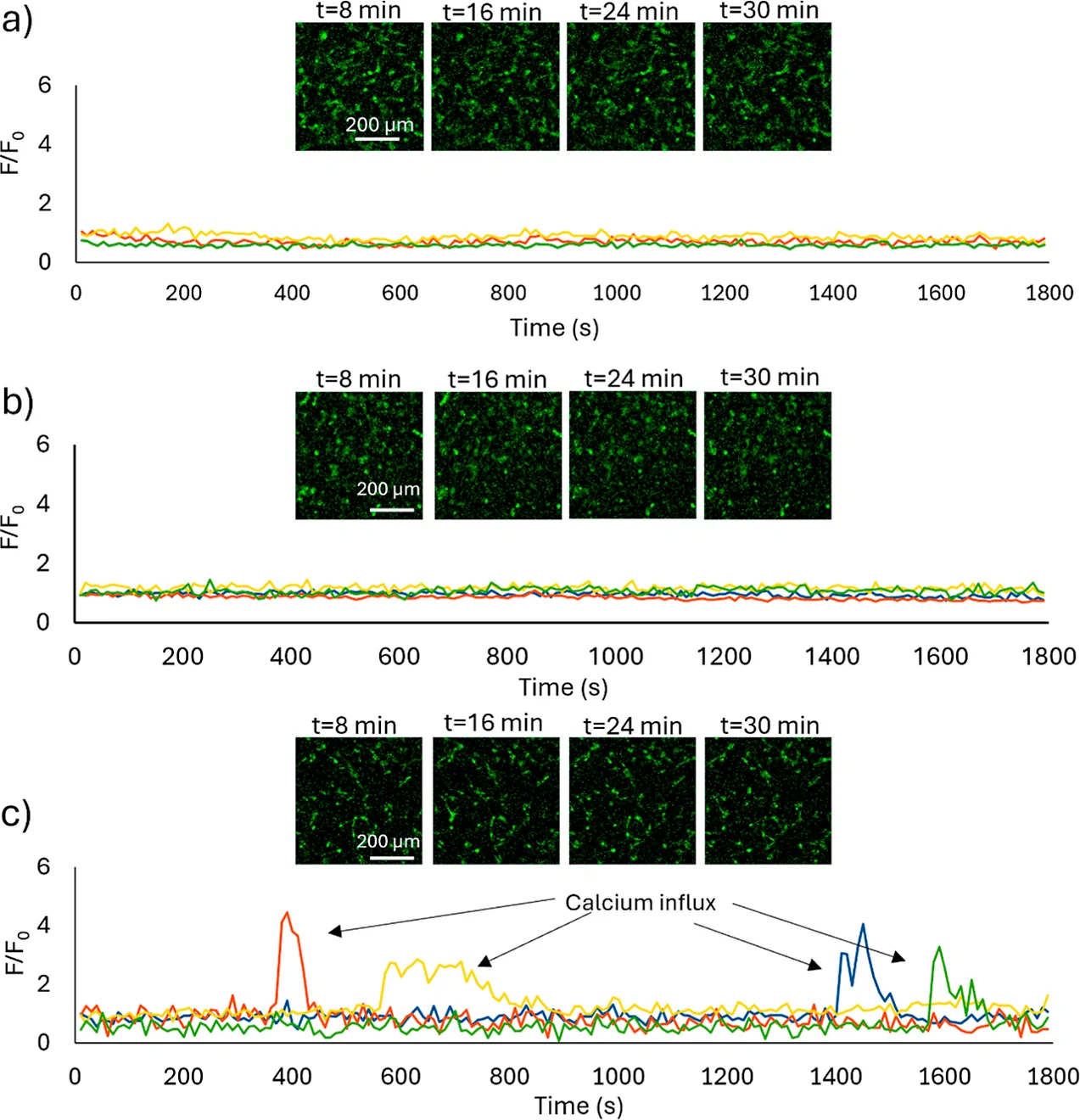
Calcium imaging following US stimulation of U87 MG cells for (a) plain US, (b) US + PDA-NPs, and (c) US + PDA-NTs. Representative confocal images (top) and quantitative analysis (bottom). Black arrows represent fluorescence intensity spikes corresponding to increases in intracellular calcium levels.

### Release of Bioactive Dopamine

DA release from PDA-NTs was quantified both in presence and absence of US stimulation (Figure 7a): at 24 h, no significant differences were observed between the two experimental groups; however, after 48 h, a slight increase in the release of free DA was observed in the presence of US stimulation (77.0 ± 18.9 μg/mL, while 61.8 ± 3.0 μg/mL for the control). After 72 h, the concentration of free DA in the supernatant reached 96.3 ± 2.6 μg/mL in the US group, while 65.2 ± 4.0 μg/mL for the control. High-performance liquid chromatography (HPLC) analysis of the collected supernatants (Figure S17) revealed a complex chromatographic profile characterized by multiple peaks, indicating that, in addition to free DA, other species are present, likely arising from PDA degradation and/or intermediate products. Notably, while the peak corresponding to DA was present at each measured time point, additional peaks also persisted, suggesting a progressive and heterogeneous process rather than the exclusive release of the monomer.

**Figure 7:**
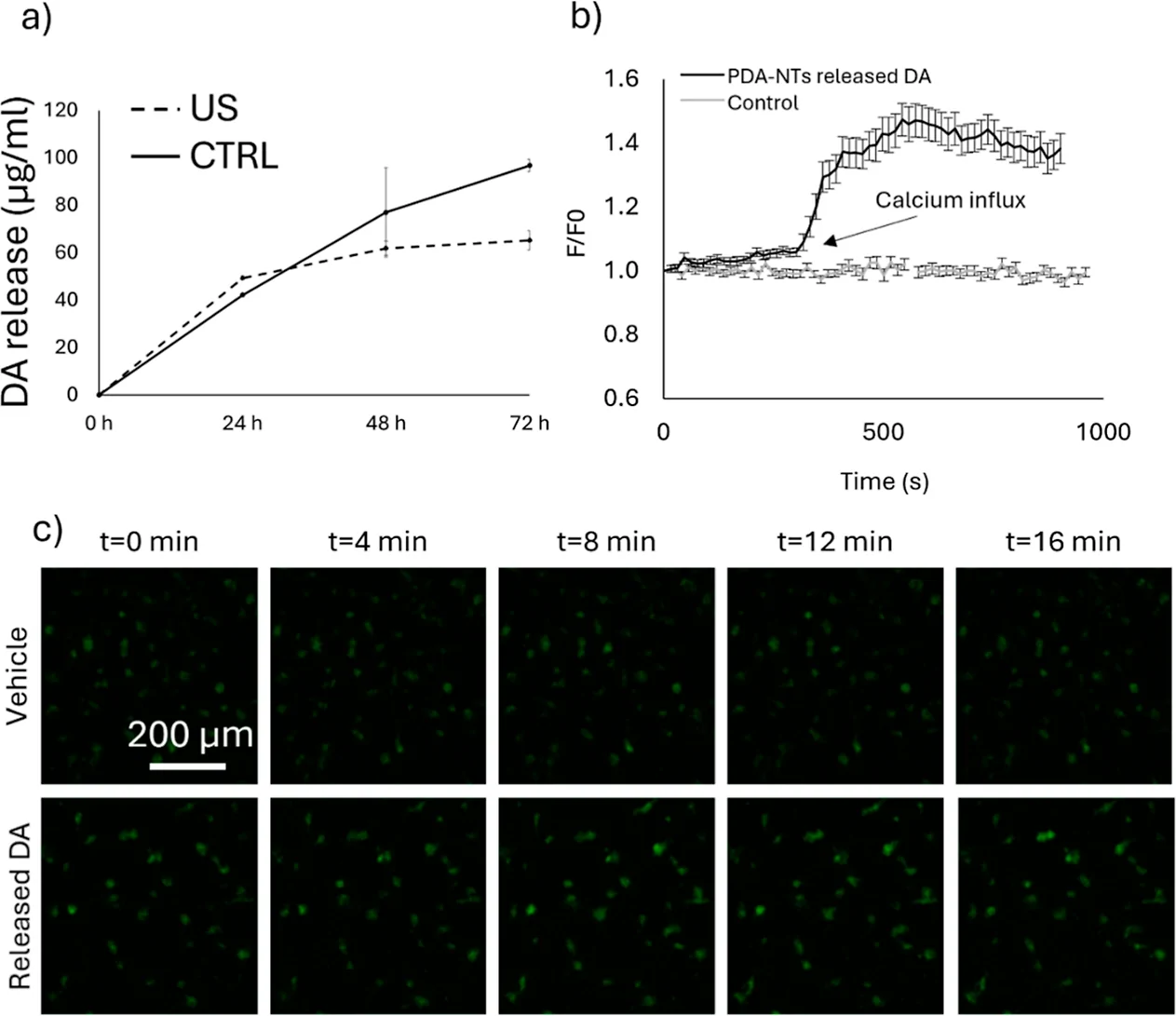
(a) DA release profile from PDA-NTs with and without US stimulation. (b) quantitative analysis of calcium imaging performed on hNSCs upon administration of DA released from PDA-NTs. Control vehicle-treated cultures are shown as well. (c) representative confocal images.

Upon the administration of released DA to hNSCs (final concentration 25 μM, obtained from US-stimulated PDA–NTs), calcium imaging was performed on the cultures. A robust increment in intracellular fluorescence intensity was observed (quantitative analysis in Figure 7b, while representative confocal images of the time-lapse in Figure 7c). Evaluation of normalized fluorescence signals *(F/F*_*0*_) revealed a progressive increase in calcium levels with respect to the baseline (at *t* = 0) in the presence of released DA. Conversely, vehicle-treated control cultures did not show any appreciable fluctuations (≤+5% throughout the recording).

## Discussion

The synthesis and purification of PDA-NTs were first validated through a comprehensive set of structural and compositional analyses, each of them aiming at confirming the successful removal of the template and the formation of nanotubes. Morphological imaging via SEM and TEM clearly revealed the hollow tubular structure of PDA-NTs, with an average length of ∼1 μm and an average diameter of ∼140 nm. These values are consistent with the sizes of the ZnO nanowire templates exploited in the synthesis, confirming that the nanotube architecture closely replicates the template geometry after etching. Importantly, TEM imaging of PDA-ZnO composites showed ZnO nanowires coated by PDA, while the inorganic core was no longer clearly distinguishable after the etching process, thus confirming the successful purification of PDA-NTs.

EDS and ICP analyses supported the extensive removal of the ZnO template. EDS spectra of PDA-ZnO showed intense Zn presence, whereas PDA-NTs displayed Zn signals close to the detection limit of EDS, supporting extensive ZnO template removal. Complementarily, ICP revealed a drastic drop in zinc content from ∼76.0% (w/w) in PDA-ZnO to just ∼1.5% (w/w) in PDA-NTs. This residual level suggests that only trace Zn-containing species remain after etching, further confirming the effectiveness of the NH_4_Cl etching process. TGA further supported these data. Pristine ZnO nanowires displayed a sharp decomposition peak at ∼256 °C, attributed to the loss of bound water and hydroxide impurities. In PDA-ZnO, the peak shifted slightly to ∼267 °C, suggesting partial stabilization by the PDA shell. In contrast, PDA-NTs did no exhibit such peak, instead showing a broad and gradual weight loss typical of organic polymer decomposition.^30^ The loss of the sharp ZnO-related peak strongly supports extensive removal of the inorganic core and the successful production of a purely organic nanotubular structure.

XRD analysis confirmed these findings at the crystallographic level. Pristine ZnO exhibited sharp, well-defined peaks characteristic of the wurtzite phase, confirming its high crystallinity.^31^ These reflections were not detectable in PDA-NTs after etching; instead, a broad halo centered around 2θ = 25° was observed, consistent with the amorphous structure of PDA. The absence of any residual ZnO peaks indicates not only complete template removal but also high structural purity, distinguishing PDA-NTs from hybrid or partially etched composites.^32^

Raman spectroscopy provided additional confirmation of the successful removal of the ZnO template. ZnO nanowires exhibited a characteristic vibrational peak at 438 cm^–1^, indicative of high crystallinity, which persisted in PDA-ZnO but was no longer detectable in PDA-NTs.^32^ Conversely, two peaks at 1348 cm^–1^ (D band) and at 1594 cm^–1^ (G band) were present in PDA-NTs and PDA-ZnO but were absent in pristine ZnO.^29^ These bands correspond to the disordered domains of PDA, further confirming the transition from crystalline inorganic template to amorphous organic nanotubes.^28^ The combination of these Raman signatures provides both a “negative control” (loss of ZnO peak) and a “positive control” (presence of PDA-specific peaks), strengthening the structural assignment.

DLS and ζ-potential analysis offered complementary information on the hydrodynamic size and the surface charge of PDA-NTs in aqueous suspension. DLS revealed a hydrodynamic size of ∼1.1 μm, consistent with TEM measurements. The ζ-potential of ∼ −16.5 mV indicated a moderately negative surface charge, typical of PDA structures,^11,15–18^ that promotes colloidal stability and is favorable for nanostructure biocompatibility. However, it should be noted that DLS assumes spherical particles and reports an equivalent hydrodynamic size, which may not accurately represent the true length or aspect ratio of nanotubes.^33^

Altogether, this multitechnique characterization provides uniquely rigorous confirmation of successful PDA-NT synthesis. Unlike prior studies that reported PDA-NTs primarily for catalysis or drug delivery, where characterization was limited to morphology, we systematically combined structural, compositional, thermogravimetric, spectroscopic, and colloidal analyses.^23–25,34^ This complete characterization strongly supports extensive removal of the ZnO template, validating the reproducibility of the synthesis process.

The discovery of strong piezoelectric behavior in PDA-NTs represents one of the most novel aspects of this study. Using PFM, we observed a significant piezoelectric coefficient (*d*_33_ ∼15 pC/N) in PDA-NTs. This value not only exceeds that one of the ZnO nanowire templates (∼6 pC/N), but is also remarkable for an organic polymer-based material. As shown in Table 1, the *d*_33_ value of PDA-NTs exceeds that reported for other commonly used inorganic and organic nanomaterials, including boron nitride (BN), polyhydroxybutyrate (PHB), gallium nitride (GaN), poly-l-lactic acid (PLLA), nylon-11, and aluminum nitride (AlN). Conversely, spherical PDA-NPs displayed no detectable piezoresponse, highlighting that piezoelectricity is not an intrinsic property of PDA, but an emergent property conferred by nanotubular morphology. The origin of this morphology-dependent behavior may be related to the molecular organization within PDA-NTs. PDA is a heterogeneous polymer containing catechol, amine, and indole-like units capable of forming hydrogen bonds, π–π stacking, and dipolar interactions.^35^ In isotropic PDA-NPs, these interactions are randomly distributed, leading to local dipole cancellation. By contrast, the anisotropic tubular geometry of PDA-NTs may impose directional constraints during polymerization around the ZnO template, favoring partial alignment of dipoles along the tube axis. Once the ZnO template is removed, this aligned organization may be preserved, potentially contributing to the observed piezoresponse. The hollow architecture may further amplify electromechanical coupling, as curvature and asymmetry can enhance strain-induced polarization effects.^36^

**Table 1:** Average *d*_33_ Values of Different Materials in Comparison with PDA-NTs

material	**d**_33_ (pC/N)	reference
boron nitride—BN nanotubes)	0.3	^76^
polyhydroxybutyrate—PHB (bulk)	2.1–2.5	^77,78^
gallium nitride—GaN (bulk)	2.0–4.0	^79,80^
poly-L-lactic acid—PLLA (nanofibers)	3.1	^79,81^
nylon-11 (bulk)	3.8–4.0	^82,83^
aluminum nitride—AlN (bulk)	3.0–6.0	^79,80,84^
polyvinylidene fluoride—PVDF (bulk)	–20.0 – −33.0	^85^
zinc oxide—ZnO (bulk)	3.0–20.0	^86,87^
PDA-NTs (nanotubes)	14.3–15.7	N/A
lithium niobate—LiNbO_3_ (single crystal)	16.0–41.5	^79,88^
polyvinylidene fluoride-trifluoroethylene—PVDF-TrFE (bulk)	21.5–74.0	^89,90^
lead zirconate titanate—PZT (bulk)	225.0–590.0	^79,91^
Potassium–sodium niobate—KNN (bulk)	93.0–700.0	^92,93^
barium titanate—BaTiO_3_ (nanoparticles)	90.0–788.0	^94^

Although this interpretation is consistent with experimental observations, the molecular and structural determinants underlying the observed electromechanical response remain to be fully elucidated. Further theoretical, computational, and structural investigations will be valuable for determining whether the nanotubular architecture promotes preferential organization of polar domains within PDA and whether this organization is sufficient to account for the measured electromechanical response. Quantitative modeling of the relation among nanotube geometry, molecular arrangement, and piezoresponse could provide additional insight into the physical origin of the observed behavior.

Interestingly, the piezoelectric coefficient of PDA-NTs is more than twice that of their ZnO nanowire templates. This is unexpected, as ZnO is a well-established inorganic piezoelectric material, while PDA has no reported piezoelectric activity.^37^ This suggests that PDA-NTs may acquire an enhanced electromechanical response through shape- and assembly-driven phenomena. One possible explanation is that the polymer matrix allows for greater deformation under applied electric fields compared to rigid crystalline ZnO, resulting in larger measurable displacements. Moreover, the heterogeneous distribution of functional groups in PDA may create microdomains of polarity that, when organized in an anisotropic framework, contribute synergistically to the overall piezoresponse. From a broader perspective, these findings demonstrate that nanostructure morphology can be a critical determinant of emergent material properties. Similar shape-induced piezoelectricity has been reported in specific peptide-based nanostructures and organic ferroelectrics, but to the best our knowledge, this is the first report of such behavior in PDA.^38–40^

Biocompatibility is one of the main properties of PDA, and our findings confirm that nanotubular morphology maintains this essential property.^1,8,11,14,16–18^ Viability assays in U87 MG cells demonstrated that PDA-NTs did not induce significant cytotoxicity at concentrations up to 100 μg/mL, as reflected by stable proportions of viable and necrotic populations. This aligns with previous studies on PDA-NPs, which consistently reported low toxicity and high cellular tolerance across diverse cell types.^1,8,11,14,16–18^ The preservation of biocompatibility in PDA-NTs is critical, as changes in morphology or surface chemistry often alter nanoparticle–cell interactions in unpredictable ways: for example, it has been demonstrated that the aspect ratio, the morphology, and the dimensions of nanostructures can play key roles in determining biocompatibility, cellular uptake, and drug release.^41,42^ The absence of adverse effects confirms that the transition into a nanotubular structure does not compromise the inherent biocompatibility of PDA.

Cellular imaging provided insight into how PDA-NTs interface with biological systems. Confocal microscopy showed clear internalization after 24 and 72 h of exposure, with partial colocalization within lysosomes. The lysosomal pathway is a common fate for endocytosed nanoparticles, and its observation here indicates that PDA-NTs follow established endocytic routes rather than inducing atypical or harmful trafficking behaviors.^8,14,17,18^ Notably, Pearson’s correlation coefficients (∼0.26–0.28) revealed only partial lysosomal colocalization, suggesting that a fraction of PDA-NTs either remain in the cytosol or are associated with other organelles. Such distribution may be advantageous for multifunctional applications, as it enables PDA-NTs to interact with multiple intracellular compartments rather than being entirely sequestered in degradative pathways. The observed colocalization between PDA-NTs and Cascade Blue (Pearson’s coefficient ∼0.20 ± 0.08 at 72 h) indicates involvement of nonspecific endocytic pathways, although the moderate correlation values point to a coexistence of multiple internalization mechanisms. This is consistent with the observed heterogeneous intracellular distribution, supporting that PDA-NTs are internalized through a combination of endocytic routes rather than a single dominant pathway.

SEM imaging provided complementary information, revealing PDA-NTs not only inside cells but also associated with the plasma membrane; on the cellular surface, PDA-NTs appeared as intact tubular, electron-dense structures, suggesting their morphology is preserved during interaction with cells. This structural stability is noteworthy, as many polymeric nanostructures undergo significant reshaping or collapse upon internalization.^43^ The ability of PDA-NTs to maintain their tubular form both outside and inside cells is crucial for retaining their emergent geometry-driven properties, including piezoelectricity and drug-loading potential. In comparison to previous reports of PDA-NTs synthesized for catalysis or sensing, where no biological evaluation was conducted, our findings represent the first direct evidence that PDA-NTs can stably interact with mammalian cells without adverse effects:^23–25,34^ this establishes a strong foundation for exploring their multifunctionality in therapeutic and bioelectronic contexts. Considering the anisotropic morphology and broad length distribution of PDA-NTs, it is plausible that shorter nanotubes are more efficiently internalized through endocytic pathways, whereas longer structures remain partially associated with the plasma membrane due to steric and geometrical constraints. This may explain the coexistence of intracellular and membrane-associated PDA-NTs observed by confocal microscopy and SEM imaging.

The antioxidant properties of PDA are well-known, and attributed to the redox-active catechol/quinone moieties in its polymeric backbone, which can undergo reversible oxidation–reduction cycles to scavenge ROS.^1,13–15^ In this study, we confirmed that these properties are fully preserved in the nanotubular morphology of PDA-NTs, which provide cytoprotective effects. When U87 MG cells were exposed to TBH, a strong inducer of oxidative stress, PDA-NT pretreatment significantly reduced both cytosolic and mitochondrial ROS accumulation. Flow cytometry analysis using CellROX and MitoSOX demonstrated that PDA-NTs efficiently mitigated ROS accumulation, even under high TBH concentrations. Moreover, FITC annexin V-/PI staining revealed that PDA-NTs protected cells against oxidative stress–induced apoptosis and necrosis, preserving their viability. Together, these findings provide strong evidence that PDA-NTs function not only as passive ROS scavengers, but also as active cytoprotective agents within the cellular environment.

The partial lysosomal localization observed in uptake studies may also contribute to their protective effect, as lysosomal ROS accumulation is known to trigger apoptotic pathways.^44^ By scavenging ROS in multiple subcellular compartments, PDA-NTs provide broad-spectrum protection against oxidative insults. From a biomedical perspective, this property is highly relevant: oxidative stress is indeed a central driver in several pathologies, including neurodegenerative diseases (Parkinson’s and Alzheimer’s disease), ischemic injury, chronic inflammation, and cancer progression.^45–49^ The ability of PDA-NTs to buffer oxidative stress while simultaneously offering additional functionalities (piezoelectric stimulation, photothermal conversion, drug release) makes them particularly attractive for multifunctional therapeutic strategies. For example, in glioblastoma, where ROS can both drive tumor aggressiveness and limit therapeutic efficacy, PDA-NTs could act as cytoprotective adjuncts during chemo- or radiotherapy. In neurodegenerative conditions, their antioxidant activity could help preserve neuronal viability, while their DA-release capability addresses neurotransmitter depletion.^49^

Compared to PDA-NPs, which have been extensively studied as antioxidants, PDA-NTs offer several potential advantages. First, their higher surface-to-volume ratio may enhance ROS scavenging efficiency on a *per*-particle basis; second, their hollow structure allows for the possibility of loading additional antioxidants or therapeutic molecules, creating combinatorial approaches; third, by integrating antioxidant activity with piezoelectric and photothermal functionalities, PDA-NTs provide a multimodal defense system that PDA-NPs cannot achieve. Thus, PDA-NTs represent not just an equivalent but an upgraded antioxidant nanoplatform, expanding the potential of PDA-based nanomaterials in biomedical applications.

PDA is well recognized as a broadband light absorber, in particular in the NIR window due to its eumelanin-like structure, and previous studies on PDA-NPs have demonstrated efficient photothermal conversion.^1,8,11,14–19^ In this work, we extend these observations to the nanotubular morphology, demonstrating that PDA-NTs are highly effective photothermal agents that can convert NIR light into heat in a concentration-dependent manner. Upon irradiation at 808 nm, PDA-NT dispersions exhibited temperature increases ranging from 6.0 ± 0.3 °C at 25 μg/mL to 17.0 ± 1.7 °C at 500 μg/mL, confirming their strong NIR absorption and high photothermal conversion efficiency, even higher than those of spherical PDA-NPs. Mechanistically, the photothermal effect arises from nonradiative relaxation of excited electrons in the π-conjugated structure of PDA. The hollow tubular morphology may enhance this effect in several ways. First, the increased surface-to-volume ratio facilitates efficient light absorption and heat dissipation into the surrounding medium; second, the anisotropic geometry could promote light scattering and multiple internal reflections within the hollow core, effectively extending the optical path length and amplifying absorption efficiency. These geometric contributions suggest why PDA-NTs outperform spherical PDA-NPs of similar mass in photothermal heating, consistent on previous reports.^15^ Importantly, this photothermal behavior is preserved at the intracellular level. Upon NIR irradiation, PDA-NT-treated U87 MG cells exhibited a measurable intracellular temperature increase (Δ*T* ≈ 8 °C), whereas no significant changes were observed under control conditions with plain NIR irradiation. This result demonstrates that PDA-NTs can act as effective intracellular nanoheaters, enabling localized thermal modulation within cells. Such capability is particularly relevant for triggering temperature-dependent biological responses, including modulation of ion channel activity and intracellular calcium dynamics, as observed in this study.

The biological effects of photothermal conversion were clearly demonstrated in U87 MG cells. While NIR irradiation alone did not affect intracellular calcium levels, the combination of NIR and PDA-NTs induced significant calcium influx, with approximately a 50% increase in intracellular calcium observed during the irradiation period. This indicates that PDA-NTs not only generate localized heating, but also transduce this physical effect into a biologically relevant signal. The most likely mechanism is thermally induced modulation of temperature-sensitive ion channels in the plasma membrane or organelles, such as transient receptor potential (TRP) channels, which are well-known to mediate heat-sensitive calcium influx.^50^ Alternatively, localized thermal stress may transiently alter membrane fluidity or permeability, indirectly facilitating calcium entry. Regardless of the precise mechanism, the data demonstrate that PDA-NTs can convert NIR light into remote modulation of intracellular signaling.^51^

The significance of this finding extends beyond proof-of-principle. Photothermal modulation of calcium signaling is of great interest for biomedical applications because calcium is a universal second messenger that regulates diverse cellular processes, including proliferation, differentiation, migration, and apoptosis. Controlled calcium activation *via* PDA-NTs could thus be exploited for therapeutic neuromodulation, cancer therapy, or regenerative medicine.^52,53^ Compared to inorganic photothermal nanomaterials such as gold nanorods, graphene, or carbon nanotubes, PDA-NTs offer several advantages: intrinsic biocompatibility, biodegradability, and the added benefit of multifunctionality (antioxidant, piezoelectric, and DA-releasing properties).^1^ This positions PDA-NTs as unique “all-in-one” photothermal platforms with superior safety and versatility.

Another important implication of the photothermal effect is the potential synergy with other functionalities of PDA-NTs. For example, localized heating could accelerate DA release from the nanotubes, enhance ROS scavenging by increasing the rates of redox reactions, or combine with piezoelectric activation for multimodal cellular stimulation. Future studies should investigate such combinatorial effects, as they could unlock unexplored therapeutic strategies that harness multiple properties of PDA-NTs simultaneously.

The piezoelectric properties of PDA-NTs were validated in a functional biological setting through US stimulation. Neither US alone nor US combined with spherical PDA-NPs elicited measurable calcium responses in U87 MG cells, confirming that acoustic waves by themselves did not alter intracellular signaling under the applied conditions. By contrast, the combination of US with PDA-NTs induced transient intracellular calcium spikes, observed as sharp fluorescence peaks during live imaging. Importantly, this response was not observed when experiments were performed in the presence of cadmium chloride, a calcium channel blocker, or in Ca^2+^-free PBS, where no significant increase in intracellular calcium levels was detected. This outcome provides compelling evidence that the electromechanical response measured by PFM is biologically relevant: PDA-NTs act as nanoscale transducers, converting mechanical acoustic energy into localized electrical stimuli that modulate ion fluxes in living cells.

One possible mechanism underlying this effect involves strain-induced polarization within the PDA-NT walls. When subjected to oscillatory pressure waves, the aligned dipolar domains of the nanotubes generate transient electric fields, which in turn influence voltage-sensitive ion channels or electrochemical gradients at the plasma membrane. The loss of calcium response under Ca^2+^-free conditions and upon cadmium chloride treatment is consistent with the hypothesis that extracellular calcium influx through membrane channels is required for this effect. The localized nature of these nanoscale fields allows for precise cellular modulation without the need for direct electrode contact.^54–57^ Importantly, this effect is absent in PDA-NPs, reinforcing the hypothesis that the emergent piezoelectricity of PDA-NTs is strictly morphology-dependent. The amplitude of the observed calcium transients is noteworthy, as they resemble physiological calcium spikes that regulate key signaling pathways.^54–57^ Unlike global and prolonged calcium elevations, transient spikes are often associated with finely tuned cellular responses such as gene expression, differentiation, and synaptic activity; this suggests that PDA-NT-mediated piezoelectric stimulation could be exploited to achieve subtle, biologically relevant modulation.^54–57^

As previously stated, confocal microscopy and SEM observations suggest the coexistence of both intracellular and membrane-associated PDA-NT populations. While a fraction of PDA-NTs is effectively internalized through endocytic pathways, longer nanotubes may remain partially associated with the plasma membrane due to steric and geometrical constraints. In the context of US stimulation, these membrane-associated PDA-NTs are likely particularly relevant for the observed extracellular Ca^2+^ influx, as their close interaction with the cell membrane may facilitate the modulation of calcium-permeable ion channels through localized piezoelectric effects.

The use of US as a stimulus offers significant translational advantages:^54–58^ US is already widely used in clinical practice for both diagnostic imaging and therapeutic interventions, and its ability to penetrate deep tissues noninvasively makes it ideal for remote control of implanted or systemically delivered nanomaterials. Traditional inorganic piezoelectric nanostructures, such as barium titanate or ZnO nanowires, have been explored for similar purposes, but they suffer from drawbacks including poor biodegradability, potential toxicity, and limited biocompatibility.^59^ In contrast, PDA-NTs combine robust piezoelectric functionality with the intrinsic safety and versatility of an organic, bioinspired polymer, offering a much more favorable risk–benefit profile for biomedical applications.

The demonstration of US-driven piezoelectric activity in PDA-NTs also opens exciting opportunities for neuromodulation. Neural activity is tightly regulated by electrical and calcium signaling, and the ability to remotely modulate these processes with biocompatible nanotransducers could provide noninvasive alternatives to electrode-based stimulation.^60^ Beyond the nervous system, PDA-NT-mediated piezoelectric stimulation may be valuable in controlling mechanosensitive pathways in stem cells, cardiomyocytes, or cancer cells, where calcium signaling plays critical roles in differentiation, contraction, and proliferation, respectively.^61,62^

Although the combination of PFM measurements, morphology-dependent controls, and ultrasound-mediated cellular responses consistently supports a piezoelectric interpretation, the present study was not designed to establish the origin of the observed electromechanical response. Future studies integrating theoretical predictions with quantitative experimental validation will be valuable to further clarify the physical basis of this phenomenon.

Taken together, these findings establish PDA-NTs as a rare example of an organic nanostructure capable of mechanically induced, electrically mediated modulation of cellular activity. This not only validates the emergent piezoelectricity of PDA-NTs in a living system, but also highlights their potential as safe and versatile tools for remote bioelectronic interfaces and precision therapy.

Beyond their physical and chemical multifunctionality, PDA-NTs demonstrated a remarkable capacity to act as reservoirs of bioactive neurotransmitters. DA, the monomer from which PDA is formed, remains partially trapped within the polymeric network of the nanotubes during synthesis. This residual DA can be gradually released into the surrounding medium, both through passive diffusion and under externally triggered conditions such as US stimulation. Another possible mechanism involves the gradual degradation of the PDA nanotube walls themselves, leading to the breakdown of the polymeric matrix and a consequent release of DA monomers: such degradation may be accelerated under specific conditions, such as prolonged US exposure.^63^

HPLC confirmed the presence of free DA in supernatants collected from PDA-NT suspensions. Importantly, DA release followed two distinct profiles: (i) a baseline passive release, likely governed by slow desorption or hydrolysis of unpolymerized DA residues, and (ii) a markedly enhanced release upon periodic US stimulation. The latter likely arises from the piezoelectric deformation of the PDA-NT walls under acoustic stress, which transiently increases the polymer porosity and facilitates the diffusion of encapsulated molecules.^64^ This dual passive/triggered release mechanism is advantageous because it combines continuous background supply with the possibility of temporally precise and remote-controlled delivery.

The biological activity of the released DA was validated using hNSCs. Exposure of hNSCs to DA-containing supernatants induced calcium signaling responses, a hallmark of neurotransmitter functionality;^53^ conversely, no effects were observed on vehicle-treated cells. These findings confirm not only that DA is released, but also that it retains its structural integrity and neuroactive properties after release from PDA-NTs. This establishes PDA-NTs as functional neuromodulatory agents rather than inert drug carriers, with important implications for biomedical applications.

HPLC analysis further indicates that the released species are not limited to the DA monomer, but rather include a mixture of low-molecular-weight oligomeric products, likely arising from partial PDA depolymerization or oxidation processes. In particular, the presence of multiple chromatographic peaks suggests a distribution of species with increasing molecular weight, which can tentatively be attributed to DA oligomers (e.g., dimers, trimers, and higher-order species), as previously reported for polydopamine degradation and intermediate formation.^65^ Such oligomeric species are known to rapidly form due to the intrinsic reactivity of catechol groups, which undergo oxidation and subsequent polymerization, making their isolation and use as analytical standards experimentally challenging. Although a definitive assignment of each chromatographic peak is not currently feasible, due to the instability and rapid oxidation of dopamine oligomers, their identification could in principle be achieved through LC–MS analysis, which would allow correlation of retention times with molecular masses. Future studies employing HPLC coupled to mass spectrometry will therefore be essential to fully elucidate the molecular composition of the released species and to discriminate between monomeric and oligomeric contributions.

This notwithstanding, despite this chemical heterogeneity, the observed calcium signaling responses in hNSCs indicate that the bioactive fraction, likely dominated by free DA or rapidly dissociating oligomeric species, remains functionally effective. In the context of Parkinson’s disease, where DA depletion in the striatum underlies the majority of motor symptoms, PDA-NTs could thus provide a unique strategy for neurotransmitter replacement.^66,67^ Conventional DA therapies, such as l-DOPA administration, are limited by systemic side effects, poor temporal control, and progressive loss of efficacy.^67^ By contrast, PDA-NTs could be locally delivered and act as on-demand reservoirs, releasing DA in response to external US triggers that can be applied noninvasively and with spatial precision. This approach may more closely mimic physiological DA fluctuations than continuous pharmacological dosing, potentially improving therapeutic outcomes and reducing side effects.

Beyond Parkinson’s, PDA-NT-mediated DA release could be harnessed in other contexts where dopaminergic signaling plays a role, including neurogenesis, mood regulation, and reward pathways.^68–70^ The demonstration that DA released from PDA-NTs can directly modulate calcium signaling in hNSCs also suggests applications in regenerative medicine, where controlled dopaminergic stimulation may guide differentiation or maturation of neuronal precursors.

When compared to conventional nanocarriers, PDA-NTs offer several unique advantages. Unlike polymeric or liposomal drug delivery systems, where cargo is typically loaded exogenously,^71,72^ PDA-NTs inherently contain DA due to their synthesis procedure, reducing the need for additional encapsulation steps. Moreover, while most drug carriers rely only on passive release or environmental triggers (e.g., pH), PDA-NTs integrate passive baseline release with remote control *via* US stimulation. This multifunctional release strategy, combined with their photothermal, antioxidant, and piezoelectric properties, makes PDA-NTs one of the most versatile neurotransmitter delivery systems described to date: by combining neuromodulatory drug delivery with physical stimulation modalities, PDA-NTs bridge the gap between nanomedicine and bioelectronics, offering a platform with transformative potential for neurological disorders and beyond.

## Conclusions

This study establishes PDA-NTs as a unique class of multifunctional organic nanostructures with emergent, geometry-driven properties. Through comprehensive physicochemical and biological analyses, we demonstrated that PDA-NTs integrate four distinct functionalities within a single platform: (i) a robust electromechanical response suggestive of a piezoelectric mechanism, (ii) strong photothermal conversion abilities, (iii) powerful antioxidant activity, and (iv) controlled release of bioactive DA, both passively and in response to US stimulation. Together, these properties highlight PDA-NTs as versatile and biocompatible multifunctional nanodevices with significant therapeutic potential.

The emergence of electromechanical transduction properties exclusively in tubular morphology highlights the critical role of structural anisotropy in determining the functional properties of organic polymers. While the present results consistently support a piezoelectric interpretation, the origin of the observed response remains to be fully elucidated and will benefit from future theoretical and quantitative experimental investigations. Conversely, spherical PDA-NPs lacked electromechanical conversion features, and failed to mediate US-driven calcium influx, underscoring that morphology is not merely a structural parameter but a key determinant of the physicochemical properties and bioactivity of PDA-based nanostructures. Moreover, the multifunctionality of PDA-NTs extends beyond previous reports on PDA-based nanomaterials, which have primarily focused on drug delivery or photothermal therapy. Here, we demonstrate that PDA-NTs can actively modulate intracellular signaling, protect cells from oxidative stress, and deliver neuromodulatory molecules—all within the same nanoplatform.

Looking forward, several points should be investigated to translate these findings into practical applications. In vivo validation: while biocompatibility and functionality were confirmed in vitro, systematic studies in mammalian models are required to assess biodistribution, clearance, long-term safety, and the ability to modulate cellular activity in living tissues. *Scalable synthesis and reproducibility*: achieving consistent nanotube sizes, piezoelectric alignment, and batch-to-batch reproducibility will be critical for clinical translation and regulatory approval. Optimizing template-based fabrication or developing template-free synthesis routes may improve scalability. *Integration into devices and scaffolds*: embedding PDA-NTs into hydrogels, tissue scaffolds, or implantable bioelectronic devices could unlock new therapeutic opportunities in regenerative medicine, neuromodulation, and oncology. *Synergistic exploitation of PDA-NT properties*: future studies should explore how PDA-NT functionalities can be combined into a single therapeutic strategy. For example, NIR-triggered DA release, US-mediated neuromodulation under antioxidant protection, or combined photothermal–piezoelectric stimulation could represent innovative approaches to tackle biomedical needs. *Expansion beyond DA*: while DA release was a natural outcome of PDA chemistry, the hollow architecture of PDA-NTs could be exploited to load and release a wide range of therapeutic agents, broadening their applicability beyond neurological disorders.

In summary, PDA-NTs represent more than a morphological variant of PDA nanostructures: they are a multifunctional nanoplatform with emergent properties uniquely enabled by nanotubular geometry. The convergence of morphology-dependent controls, PFM measurements, and ultrasound-mediated biological responses provides strong experimental evidence supporting a piezoelectric mechanism associated with this architecture. Their demonstrated ability to interface with cells through physical, chemical, and biochemical pathways opens new horizons in precision medicine, neuroengineering, oncology, and advanced bioelectronics. With further optimization and translational studies, PDA-NTs could become a cornerstone of next-generation multifunctional nanotherapeutics.

## Materials and Methods

### Synthesis of Polydopamine Nanotubes

PDA-NTs were fabricated using a combination of the Stöber reaction and a template removal strategy. ZnO nanowires (PlasmaChem, 50–80 nm in diameter, 0.2–7.0 μm in length) served as the template for PDA-NT formation. Specifically, 80 mg of ZnO nanowires were dispersed in 40 mL of TRIS–HCl buffer (10 mM, pH 8.5) *via* sonication (Fisherbrand Q125 Sonicator, 80 W for 5 min on ice) and maintained under magnetic stirring at 1500 rpm. Subsequently, 40 mg of dopamine hydrochloride (Sigma), predissolved in water (final concentration 100 mg/mL), was added to the ZnO nanowire dispersion, and the mixture was stirred for 24 h. After this incubation, the resulting PDA-ZnO were purified through 3 steps of centrifugation at 12,000*g* for 10 min at 4 °C; each time the pellet was resuspended in water and sonicated for 1 min at 40 W. Following the final wash, the PDA-ZnO structures were treated with 2 M NH_4_Cl under stirring at 60 °C for 1 h to dissolve the ZnO template. The resulting PDA-NTs were then purified via centrifugation at 30,000*g* for 20 min at 4 °C, repeated 5×. Each time the obtained pellet was resuspended in Milli-Q water and sonicated for 1 min at 40 W. Finally, the purified PDA-NTs were quantified by freeze-drying.

PDA-NPs of approximately 1 μm in diameter, exploited as negative controls in some of the experiments described in the following, were fabricated as described in previous works from our group.^15^

### Physicochemical Characterization of Polydopamine Nanotubes

The hydrodynamic size distribution and zeta potential (ζ-potential) of PDA-NTs were measured using a Malvern Zetasizer Nano ZS90 (Malvern Instruments). For all DLS measurements, freshly prepared PDA-NT suspensions were diluted in Milli-Q water to a final concentration of 100 μg/mL to minimize scattering effects. Disposable polystyrene cuvettes were used for size measurements, whereas folded capillary cells were exploited for ζ-potential measurements. The average hydrodynamic size and ζ-potential were derived by the instrument software (Malvern Zetasizer Software v7.12) based on the cumulant analysis of the autocorrelation function and on electrophoretic light scattering.

For SEM analysis, water-dispersed samples (ZnO nanowires, PDA-ZnO, PDA-NTs, and PDA-NPs, at a concentration of approximately 10 μg/mL) were drop-cast on a fragment of a silica wafer and air-dried. Thereafter, the samples were gold-sputtered using a Quorum SC7620 mini–Gold Sputter Coater (15 mA for 30 s). Images of the samples were obtained exploiting a dual-beam SEM system (Helios NanoLab 600i FIB/SEM, FEI).

For the negative-staining TEM analysis, the samples (PDA-ZnO and PDA-NTs) were diluted to approximately 0.1 mg/mL in Milli-Q water. Then, they were drop-cast onto ultrathin C 150 mesh Cu grids and stained with 1% uranyl acetate in water for 60 s before being analyzed with a JEM-1011 TEM (JEOL) equipped with a thermionic source (W filament) operating at 100 kV. Images were acquired with an Emsis TOLARA B06U series CCD camera (2752 × 2192 active pixels).

The same samples, deposited on grids, were analyzed using a high-resolution SEM (HRSEM) with a Zeiss GeminiSEM 560 equipped with a field-emission gun operating at an acceleration voltage of 10 kV. EDS (Oxford Instruments, X-Max, 80 mm^2^) operating at 10 kV was used to evaluate the presence of Zn, C, and O. For EDS analysis on higher surface areas, a benchtop Phenom XL G2 SEM was exploited. Samples were prepared as previously described; PDA-NTs subjected to 15, 30, and 60 min of etching in NH_4_Cl were analyzed.

TGA was performed using a TA Instruments Q500 thermal analyzer under a nitrogen flow of 40 mL/min on approximately 5 mg of sample powder (ZnO nanowires, PDA-ZnO, and PDA-NTs). After a 5 min equilibration at 30 °C, the temperature was increased at a rate of 5 °C/min to 600 °C.

PDA-ZnO and PDA-NTs were further analyzed using an inductively coupled plasma optical emission spectrometer (ICP-OES), model iCAP 7600DUO (Thermo Fisher Scientific). An aliquot of 20 mg of the samples was accurately weighed and digested in 1 mL of freshly prepared *aqua regia*. The mixture was allowed to react for 8 h at room temperature. After digestion, the solution was diluted with Milli-Q water to a final volume of 10 mL before ICP analysis.

XRD analysis was carried out on ZnO nanowires and PDA-NTs, by exploiting a Malvern-PANalytical third-generation Empyrean X-ray powder diffractometer. The instrument was equipped with a 1.8 kW CuKα X-ray tube operating at 45 kV and 40 mA, equipped with a iCore and dCore automated PreFIX beam optical modules and a PIXcel3D 2 × 2 area detector. The XRD patterns were collected in air at room temperature in Bragg–Brentano geometry with the detector set in 1D mode.

Raman spectra were acquired to evaluate the vibrational features of ZnO nanowires, PDA-ZnO, and PDA-NTs. Measurements were performed using a Horiba LabRAM HR Evolution Raman microscope equipped with a 532 nm laser and a 100× objective. Each spectrum was collected over a spectral range of 400–1800 cm^–1^ with an acquisition time of 5 s and averaged over 10 accumulations to improve the signal-to-noise ratio. All samples were deposited on CaF_2_ substrates and allowed to dry, then analyzed under identical experimental conditions.

Surface chemical composition and the presence of specific functional groups were further investigated using XPS (Kratos, Axis UltraDLD). Both survey spectra and high-resolution spectra were acquired using a monochromatic Al Kα source operated at 20 mA and 15 kV. Survey spectra were acquired at a pass energy of 160 eV, an energy step of 1 eV, and over an analysis area of 300 × 700 μm. High-resolution spectra were acquired on the same area, at a pass energy of 20 eV and with an energy step of 0.1 eV. The Kratos charge neutralizer system was used on all samples; binding energy scale calibration was performed by setting the position of the main C 1 s component at 284.5 eV (for C=C bonds). Casa XPS software was used to analyze experimental data. The samples were deposited on an indium substrate, PDA-NTs subjected to 15, 30, and 60 min of etching in NH_4_Cl were analyzed.

AFM and PFM characterization was conducted using an Oxford Instruments-Asylum Research MFP-3D device, equipped with a PFM module. ZnO nanowires, PDA-ZnO, PDA-NTs, and PDA-NPs (water-dispersed at a final concentration of approximately 10 μg/mL) were drop-cast on a fragment of a silica wafer and air-dried. The measurements were carried out under controlled environmental conditions (temperature 30.0 ± 0.1 °C, relative humidity <5%) by exploiting Asylum AC240-R3 conductive cantilevers (Electrilever, Ti/Ir-coated, with a nominal spring constant of 2 N/m and a free resonance frequency of approximately 70 kHz). The mechanical parameters of the cantilever and the optical lever sensitivity were calibrated using the contactless method provided by the instrument supplier (GetReal). To assess the *d*_33_ constant, PFM was performed in off-resonance mode by applying an AC signal at approximately 300 kHz with an amplitude of 10 V. The synchronous deformation A was detected as vertical deflection. Under these off-resonance conditions, the piezoelectric constant was calculated as *d*_33_ *= A/V*_ac_.

To improve measurement sensitivity and statistical reliability, contact-resonance piezoresponse measurements were performed. Measurements were acquired with an AC excitation amplitude of 1 V at a modulation frequency of 301 kHz, which corresponds to the contact-resonance frequency of the nanotube region. The contact quality factor was estimated as *Q*_contact_ = 30. The effective piezoelectric coefficient was calculated according to *d*_33_ = *A*/(*Q*_contact_ × *V*_AC_), where *A* is the measured piezoresponse amplitude, *Q*_contact_ is the contact-resonance quality factor, and *V*_AC_ is the applied AC voltage, following previously reported approaches.^73^ Because the contact-resonance parameters differ among material regions, the calculation was applied selectively to the appropriate region of interest. For this purpose, a mask was extracted from the topography image and applied to the piezoresponse data before calculating *d*_33_. The antioxidant activity of PDA-NTs and PDA-NPs was assessed using a Total Antioxidant Capacity Assay Kit (Sigma), following the manufacturer’s protocol. Nanostructures were tested at concentrations of 0, 10, 25, and 50 μg/mL for both PDA-NTs and PDA-NPs. Sample absorbance was measured at 405 nm with a Victor X3Multilabel Plate Reader (PerkinElmer), and Trolox-equivalent antioxidant capacity was calculated using a standard calibration curve. The ability of PDA-NTs to convert NIR light into heat was assessed using an RLTMDL-808–500 NIR laser (λ 808 nm, spot diameter 2.5 mm) and a thermal camera (A300, FLIR) for thermal data acquisition. Aqueous dispersions of PDA-NTs and PDA-NPs at increasing concentrations (0, 25, 50, 100, 250, and 500 μg/mL) were tested in glass-bottom 35 mm confocal Petri dishes (VWR), with the laser source set to the maximum power (541 mW). Measurements were conducted at room temperature, recording the temperature reached after 10 min of irradiation. For each experimental condition, data were collected in triplicate.

### Cell Cultures

Human glioblastoma U87 MG cells, purchased from LGC Standards (ATCC HTB-14) were cultured in Dulbecco’s modified Eagle’s medium (DMEM, Gibco) supplemented with 10% heat-inactivated fetal bovine serum (FBS, Gibco), 1% l-glutamine (200 mM stock, Gibco), and 1% penicillin–streptomycin (100 IU/mL penicillin, 100 μg/mL streptomycin, Gibco). Cells were maintained at 37 °C in a humidified atmosphere with 5% CO_2_ and routinely passaged upon reaching 70–80% confluency using trypsin-ethylenediaminetetraacetic acid, EDTA (0.05%). For all experiments, U87 MG cells were seeded at a density of 10,000 cells/cm^2^ in appropriate culture plates and allowed to adhere for 24 h before treatment. PDA-NTs were dispersed in culture medium by sonication for 10 min and administered at the desired concentration. Control groups were cultured under standard conditions without exposure to PDA-NTs. Unless otherwise stated, all treatments were carried out in phenol red-containing complete DMEM under the same incubation conditions described above.

Human neural hindbrain stem cells (hNSCs), purchased from Takara Bio Inc. (Y40060), were also exploited in some of the reported experiments. Cells were seeded at a density of 4000 cells/cm^2^ in T25 flasks precoated with laminin (10 μg/mL in PBS, 3 h at 37 °C). The cells were maintained in RHB-A medium (Takara Bio Inc.), supplemented with 100 μg/mL streptomycin (Gibco), 20 ng/mL of recombinant human epidermal growth factor (EGF, Peprotech), and 20 ng/mL of recombinant human fibroblast growth factor (FGF-2, Peprotech), under standard culture conditions. Medium replacement occurred every other day, and passaging was performed at a 1:2 ratio approximately once a week, when cultures reached 80–90% confluency. Only cells below passage six were used in the experiments.

### Biocompatibility and Internalization Tests

To assess the biocompatibility of PDA-NTs, a LIVE/DEAD assay (Thermo Fisher) was performed on U87 MG cells exposed to the nanostructures. Cells were seeded at a density of 10,000 cells/cm^2^ in 24-well Corning plates and exposed to PDA-NTs at concentrations of 0, 10, 25, and 50 μg/mL for 72 h. Following treatment, cells were rinsed with Dulbecco’s phosphate buffered saline (DPBS) and incubated for 30 min at 37 °C in phenol red-free medium containing Hoechst 33342 (5 μg/mL) to stain nuclei, ethidium homodimer-1 (4 μM) to label dead cells, and calcein-AM (2 μM) to label live cells (all from Thermo Fisher). After staining, the cells were washed with DPBS and imaged using a Nikon Eclipse Ti fluorescence microscope equipped with a 10× objective. Image analysis was performed using ImageJ software.

The intracellular distribution and uptake of PDA-NTs were examined using confocal microscopy (with a Nikon C 2s system, 60× oil immersion objective). Cells were cultured in 96-well black μ-Plates (Ibidi, cell-imaging grade) and incubated with phenol red-free medium containing 50 μg/mL PDA-NTs. After incubation, the cells were rinsed with DPBS and exposed for 30 min at 37 °C to phenol red-free medium supplemented with Lysotracker-Red (5 μM, Thermo Fisher) and Hoechst 33342 (5 μg/mL, Invitrogen) for lysosomal imaging and nucleus counterstaining, respectively. For F-actin imaging, after the incubation with PDA-NTs cells were fixed with paraformaldehyde (PFA, 4% in DPBS, 20 min at 4 °C), rinsed twice with DPBS, and incubated for 40 min in goat serum (10% in DPBS) containing TRITC-phalloidin (2.5 μg/mL, Sigma) and Hoechst 33342 (5 μg/mL, Invitrogen) at 37 °C, and then rinsed twice in DPBS. Confocal images were captured after 24 and 72 h of exposure to PDA-NTs (imaged exploiting the optical signal generated when irradiated at 642 nm).

Pinocytosis-mediated uptake of PDA-NTs was also evaluated using a protocol analogous to that described above. Briefly, cells were supplemented with 300 μM Cascade Blue hydrazide fluorescent dye (Invitrogen) and incubated with PDA-NTs (50 μg/mL) for 24 or 72 h. Following treatment, cells were imaged using a confocal microscope (Nikon C 2s system) equipped with a 60× oil-immersion objective to assess fluid-phase uptake, while PDA-NTs were visualized as previously described by exploiting fluorescence signal upon excitation at 642 nm.

Lastly, the interaction of PDA-NTs and the cell surface was analyzed through SEM imaging. U87 MG cells were plated at a density of 10,000 cells/cm^2^ on glass-bottom 35 mm confocal Petri dishes (VWR) and cultured for 24 h. The cells were then incubated with 50 μg/mL of PDA-NTs for additional 72 h. After exposure to PDA-NTs, cultures were fixed with PFA as previously described, rinsed with Milli-Q water, and postfixed with 2.5% glutaraldehyde in Milli-Q water for 2 h at 4 °C. Subsequently, cells were dehydrated through a graded ethanol series (25%, 50%, 75%, and 100%, each for 5 min), air-dried, and gold-sputtered using a Quorum SC7620 mini Gold Sputter Coater (15 mA for 30 s).

### Assessment of Polydopamine Nanotubes Antioxidant Properties

U87 MG cells were seeded at 10,000 cells/cm^2^ in 6-well plates and cultured to confluency. Cells were then treated with 50 μg/mL PDA-NTs for 72 h under standard conditions. After incubation, cells were washed with DPBS and stained while still adherent with CellROX Green reagent 5 μM (Thermo Fisher) for 30 min at 37 °C in phenol red-free medium. Following staining, cells were detached using trypsin, resuspended in phenol red-free medium, and divided into different experimental groups (CTRL, PDA-NTs, 2.5 mM TBH, PDA-NTs +2.5 mM TBH, 5.0 TBH, PDA-NTs +5.0 mM TBH). For oxidative stress induction, TBH was added to designated samples at a final concentration of 2.5 or 5.0 mM, and the suspensions were incubated for 30 min at 37 °C. Fluorescence was immediately analyzed by flow cytometry (CytoFLEX, Beckman Coulter; λ_ex_ 488 nm, λ_em_ 525 ± 40 nm) to assess ROS levels.

To evaluate the protective effect against oxidative stress-induced cell death, annexin V-FITC/propidium iodide (PI) staining was performed. After PDA-NT pretreatment (72 h), cells were exposed to TBH 100 μM or 200 μM for 24 h, detached, and stained using the FITC-annexin V/Dead Cell Apoptosis Kit (Thermo Fisher) following the manufacturer’s protocol. Samples were analyzed by flow cytometry (for FITC-annexin V λ_ex_ 488, λ_em_ 525 nm; for PI λ_ex_ 488, λ_em_ 610 nm) to quantify viable, apoptotic (FITC-annexin V-positive), and necrotic (PI-positive) cell populations.

Eventually, mitochondrial superoxide levels were assessed using MitoSOX Red reagent (Thermo Fisher. After PDA-NTs treatment (72 h) and subsequent 24 h of TBH exposure (100 μM or 200 μM), cells were detached, washed, and incubated in suspension with MitoSOX Red 5 μM for 10 min at 37 °C. Fluorescence was immediately assessed by flow cytometry (λ_ex_ 488 nm, λ_em_ 580 nm) under identical instrument settings for all the groups.

### Modulation of Cellular Activity through NIR and US Stimulation

To assess intracellular temperature under NIR irradiation, U87 MG cells were seeded in glass-bottom WillCo (VWR) dishes at a density of 10,000 cells/cm^2^. After allowing cells to adhere and grow for 24 h, cultures were treated with 50 μg/mL of PDA-NTs, while control samples were maintained under identical conditions without nanomaterial exposure. Following 24 h of incubation with PDA-NTs, cells were stained with the thermosensitive fluorescent dye DiI (1,1′-dioctadecyl-3,3,3′,3′-tetramethylindocarbocyanine perchlorate, Invitrogen, 1 μM for 30 min at 37 °C). After staining, cells were washed twice in PBS and imaged with a laser-scanning confocal microscope to monitor temperature-dependent fluorescence changes. Time-lapse imaging was performed for a total duration of 160 s, consisting of an initial 60 s acquisition without NIR irradiation (baseline), followed by 60 s under continuous NIR laser exposure and a final period of 40 s without NIR irradiation. Fluorescence intensity variations were used as an indirect measure of intracellular temperature changes, according to a previous work from our group.^74^

For the analysis of intracellular Ca^2+^ variations under NIR irradiation, U87 MG cells were seeded in glass-bottom 35 mm confocal Petri dishes (VWR) at 4000 cells/cm^2^. After 24 h from the seeding, cultures were treated with 50 μg/mL of PDA-NTs (control cultures were performed as well). After additional 24 h of incubation, cells were stained with Fluo-4 AM (1 μM, Invitrogen) for 40 min at 37 °C. Samples were then rinsed with DPBS (Sigma) and incubated in HEPES-supplemented (25 mM) phenol red–free DMEM (Thermo Fisher) for time-lapse laser scanning confocal microscopy (Nikon C 2s system). Control and PDA-NTs-treated cultures were irradiated with a NIR laser (λ 808 nm, RLTMDL-808–500) using a 2.5 mm spot diameter and 532 mW power for 24 min. Images were acquired from three independent fields every 15 s with a 10× objective. Fluorescence intensities were calculated by averaging pixel values within the intracellular region of interest (ROI). Baseline fluorescence intensity, denoted as *F*_*0*_, was measured at time *t* = 0 s, while intensities *F* were measured at following time points. *F/F*_*0*_ ratios were plotted for both experimental conditions (NIR and NIR + PDA-NTs) throughout the time-lapse experiment. Representative images from both experimental groups were selected at defined time points to show the Ca^2+^ flux over time.

For the assessment of cell piezoelectric stimulation, U87 MG cells were seeded in 24-well μ-Plates (Ibidi) at a density of 3000 cells/cm^2^. Three experimental groups were set: one receiving US stimulation in standard culture medium (US), one receiving US in the presence of PDA-NPs, (US + PDA-NPs) and the last one receiving US in the presence of PDA-NTs (US + PDA-NTs). At 24 h postseeding, PDA-NPs were added to the US + PDA-NP group at a final concentration of 50 μg/mL, while PDA-NTs were added to the US + PDA-NTs group at the same final concentration; the medium in the US group was refreshed to maintain consistency. After an additional 24 h, cells were stained with Fluo-4 AM (1 μM, Invitrogen) for 40 min at 37 °C. Following incubation, cells were rinsed with PBS and placed in phenol red-free DMEM with HEPES (25 mM, Thermo Fisher) for time-lapse fluorescence imaging using a Nikon Eclipse Ti-E confocal microscope. Ca^2+^ flux was monitored for 30 min through confocal imaging (10× objective, one image every 10 s). US stimulation was initiated 5 min after the start of the time lapse and was applied as previously described^54–57^ using a KTAC-4000 device (Sonidel) connected to a planar US transducer (20 mm diameter), set to a power density of 1 W/cm^2^, a frequency of 1 MHz, a burst rate of 0.5 Hz, and a duty cycle of 10%. These settings were selected to minimize nonspecific mechanical and thermal stimulation of the cells, based on previous results from our group.^75^ As previously described, fluorescence intensities were calculated by averaging pixel values within the intracellular ROI, and *F/F*_*0*_ ratios were plotted to illustrate fluorescence traces under all experimental conditions (US, US + PDA-NPs, and US + PDA-NTs) throughout the time-lapse experiment. Representative images from all experimental groups were selected at defined time points to show the Ca^2+^ flux over time.

Additional calcium imaging experiments were performed to investigate the mechanisms underlying Ca^2+^ influx during US stimulation. In one condition, cells were stimulated following the same protocol described above (US applied from 5 to 25 min) in the presence of cadmium chloride (CdCl_2_, 10 μM), a known blocker of voltage-gated calcium channels. In a second condition, cells were incubated in calcium- and magnesium-free PBS to evaluate the contribution of extracellular calcium to the observed responses. In both cases, cells were stained with Fluo-4 AM and imaged under identical conditions as described above, and *F/F*_*0*_ traces were calculated to assess Ca^2+^ dynamics.

### Triggered Bioactive Dopamine Release

DA release experiment was conducted using PDA-NT suspensions in Milli-Q water, prepared at a concentration of 11.5 mg/mL. Two experimental groups were established: one without US stimulation (control) and one undergoing US. For each group, 2 mL of the PDA-NT suspension was placed into individual wells of a 24-well plate and incubated at 37 °C. In the US group, samples were treated with US for 1 h every 24 h using a KTAC-4000 US generator (Sonidel) with a 20 mm planar transducer. US pulse trains were delivered at an intensity of 1 W/cm^2^, a frequency of 1 MHz, a burst rate of 0.5 Hz, and a duty cycle of 10%. At specific time points (24, 48, and 72 h), 500 μL of the solution was collected from each well. Samples were then centrifuged at 4000*g* for 10 min at 25 °C (Hettich Universal 320/320R centrifuge) using Amicon Ultra 15 mL centrifugal filters with a molecular weight cutoff of 100 kDa (Ultra-4 Centrifugal Filter Unit, Sigma-Aldrich) to separate free DA from the nanotubes. The resulting supernatant was diluted 1:2 in acetonitrile, and the DA concentration was evaluated by HPLC with a Shimadzu LC-20AT system, equipped with a C-18 column (150.0 mm × 4.6 mm i.d., 5 μm particle size). The mobile phase consisted of 85% water containing 0.1% acetic acid (Carlo Erba) and 15% acetonitrile (HPLC-grade, Carlo Erba) delivered at a constant flow rate of 1 mL/min. Detection was carried out at 280 nm, with the DA peak typically eluting at approximately 2 min 45 s. Calibration was performed by plotting the peak area against known DA concentrations (in a DPBS solution supplemented with 5 μM -EDTA, Sigma-Aldrich-) to obtain a standard curve (Figure S18).

To demonstrate the bioactivity of the DA released by PDA-NTs, hNSCs were then seeded in two wells of a μ-Plate 24 Well (Ibidi) at approximately 50% confluence, with 500 μL of culture medium *per* well. Before seeding, wells were coated with 250 μL of laminin solution (10 μg/mL, 3 h at 37 °C). One well was designated for the treatment with free DA in standard culture medium, while the second served as the control group. In the experimental group treated with DA, the supernatant obtained from the PDA-NTs stimulated with US was added to the cell culture medium to achieve a final DA concentration of 25 μM. In the control group, a vehicle was added to the culture medium (Milli-Q water, 1:15 dilution), ensuring consistent dilution conditions across both experimental classes.

Calcium imaging was performed as previously described. The cells were imaged for 16 min (from three independent fields every 15 s with a 10× objective); representative images from all experimental groups were selected at defined time points to show the Ca^2+^ flux over time. Free DA and plain vehicle were added to the cell cultures after 5 min from the start of the imaging.

### Statistical Analysis

Statistical analyses were performed using *R* software. The Shapiro–Wilk test was applied to assess data normality. Thereafter, one-way ANOVA was used, followed by LSD *post-hoc* comparisons with Bonferroni’s correction to account for multiple testing. Results are presented as mean ± standard deviation. Unless otherwise noted, all experiments were performed in triplicate (*n* = 3).

## Supplementary Material







## Data Availability

All data are available from the authors upon reasonable request.

## Notes

DLS and ζ-potential analysis of PDA-NTs (**Figure S1**); SEM-EDS and XPS analyses of ZnO template removal at different etching times (**Figures S2** and **S3**); large-area AFM/PFM characterization of PDA-NTs and PFM analysis of ZnO nanowires and PDA-ZnO (**Figures S4** and **S5**); Live/Dead assay and cellular interaction/internalization analyses, including lysosomal and pinocytotic colocalization, 3D rendering, and SEM imaging (**Figures S6–S10**); antioxidant activity and representative flow cytometry analyses of cytosolic ROS, mitochondrial ROS, and apoptosis/necrosis (**Figures S11–S14**); intracellular temperature evaluation under NIR irradiation (**Figure S15**); calcium imaging under US stimulation in the presence of CdCl_2_ or calcium-free PBS (**Figure S16**); HPLC chromatogram of dopamine-related species released from PDA-NTs (**Figure S17**); HPLC calibration curve for dopamine quantification (**Figure S18**) (PDF)
Representative calcium imaging videos of U87 MG cells under US stimulation in the absence of PDA-NTs (WMV)
Representative calcium imaging videos of U87 MG cells under US stimulation in the presence of PDA-NTs (WMV)

## References

[1] Battaglini M., Emanet M., Carmignani A., Ciofani G. (2024). Polydopamine-Based
Nanostructures: A New Generation of Versatile, Multi-Tasking, and
Smart Theranostic Tools. Nano Today.

[2] Liu C.-Y., Huang C.-J. (2016). Functionalization of Polydopamine
via the Aza-Michael
Reaction for Antimicrobial Interfaces. Langmuir.

[3] Liu X., Cao J., Li H., Li J., Jin Q., Ren K., Ji J. (2013). Mussel-Inspired Polydopamine:
A Biocompatible and Ultrastable Coating
for Nanoparticles in Vivo. ACS Nano.

[4] Lee H., Dellatore S. M., Miller W. M., Messersmith P. B. (2007). Mussel-Inspired
Surface Chemistry for Multifunctional Coatings. Science.

[5] Zhang P., Xu Q., Du J., Wang Y. (2018). Polydopamine-Based Nanoparticles
with Excellent Biocompatibility for Photothermally Enhanced Gene Delivery. RSC Adv..

[6] Shimizu K., Takeoka S. (2023). Photothermal Switch of Drug Release from Polydopamine-Modified
Nanosheets. MRS Commun..

[7] Ma J., Li J., Wang X., Li M., Teng W., Tao Z., Xie J., Ma Y., Shi Q., Li B., Saijilafu (2023). GDNF-Loaded Polydopamine Nanoparticles-Based
Anisotropic Scaffolds Promote Spinal Cord Repair by Modulating Inhibitory
Microenvironment. Adv. Healthc. Mater..

[8] Emanet M., Lefevre M. C., Ceccarelli M. C., Battaglini M., Carmignani A., Schiavone F., Marino A., De Pasquale D., Prato M., De Boni F., Petretto A., Bartolucci M., Catalano F., Moscato S., Ciofani G. (2024). Polydopamine Nanoparticle-Based
Combined Chemotherapy and Photothermal Therapy for the Treatment of
Liver Cancer. ACS Appl. Mater. Interfaces.

[9] Ambekar R. S., Kandasubramanian B. (2019). A Polydopamine-Based
Platform for Anti-Cancer Drug
Delivery. Biomater. Sci..

[10] Han W., Han X., Liu Z., Zhang S., Li Y., Lu J., Chen J., Ou L., Fu G. (2020). Facile Modification
of Protein-Imprinted Polydopamine Coatings over Nanoparticles with
Enhanced Binding Selectivity. Chem. Eng. J..

[11] Carmignani A., Battaglini M., Marino A., Pignatelli F., Ciofani G. (2024). Drug-Loaded Polydopamine Nanoparticles for Chemo/Photothermal
Therapy against Colorectal Cancer Cells. ACS
Appl. Bio Mater..

[12] Li N., Wang H.-B., Thia L., Wang J.-Y., Wang X. (2015). Enzymatic-Reaction
Induced Production of Polydopamine Nanoparticles for Sensitive and
Visual Sensing of Urea. Analyst.

[13] Guo Y., Baschieri A., Mollica F., Valgimigli L., Cedrowski J., Litwinienko G., Amorati R. (2021). Hydrogen Atom Transfer
from HOO. to Ortho-Quinones Explains the Antioxidant Activity of Polydopamine. Angew. Chem., Int. Ed..

[14] Battaglini M., Carmignani A., Martinelli C., Colica J., Marino A., Doccini S., Mollo V., Santoro F., Bartolucci M., Petretto A., Santorelli F. M., Ciofani G. (2022). In Vitro Study of Polydopamine
Nanoparticles as Protective Antioxidant Agents in Fibroblasts Derived
from ARSACS Patients. Biomater. Sci..

[15] Carmignani A., Battaglini M., Sinibaldi E., Marino A., Vighetto V., Cauda V., Ciofani G. (2022). In Vitro and Ex Vivo Investigation
of the Effects of Polydopamine Nanoparticle Size on Their Antioxidant
and Photothermal Properties: Implications for Biomedical Applications. ACS Appl. Nano Mater..

[16] Battaglini M., Carmignani A., Ciobanu D. Z., Marino A., Catalano F., Armirotti A., Ciofani G. (2025). Detailed Profiling of Protein Corona
Formed by Polydopamine Nanoparticles in Human Plasma. ACS Appl. Mater. Interfaces.

[17] Carmignani A., Battaglini M., Bartolucci M., Petretto A., Prato M., Ciofani G. (2024). Polydopamine Nanoparticles
as a Non-Pharmaceutical
Tool in the Treatment of Fatty Liver Disease. Mater. Des..

[18] Battaglini M., Marino A., Carmignani A., Tapeinos C., Cauda V., Ancona A., Garino N., Vighetto V., La Rosa G., Sinibaldi E., Ciofani G. (2020). Polydopamine Nanoparticles as an
Organic and Biodegradable Multitasking Tool for Neuroprotection and
Remote Neuronal Stimulation. ACS Appl. Mater.
Interfaces.

[19] Carmignani A., Yamazaki T., Battaglini M., Vu C. Q., Marino A., Takayanagi-Kiya S., Kiya T., Armirotti A., Di Fonzo A., Arai S., Ciofani G. (2025). Cellular Activity Modulation
Mediated by Near Infrared-Irradiated Polydopamine Nanoparticles: In
Vitro and Ex Vivo Investigation. ACS Nano.

[20] Kladko D. V., Falchevskaya A. S., Serov N. S., Prilepskii A. Y. (2021). Nanomaterial
Shape Influence on Cell Behavior. Int. J. Mol.
Sci..

[21] Yasun E., Gandhi S., Choudhury S., Mohammadinejad R., Benyettou F., Gozubenli N., Arami H. (2020). Hollow Micro and Nanostructures
for Therapeutic and Imaging Applications. J.
Drug Delivery Sci. Technol..

[22] Zhang M., Li J. (2009). Carbon Nanotube in
Different Shapes. Mater.
Today.

[23] Yan D., Xu P., Xiang Q., Mou H., Xu J., Wen W., Li X., Zhang Y. (2016). Polydopamine
Nanotubes: Bio-Inspired Synthesis, Formaldehyde
Sensing Properties and Thermodynamic Investigation. J. Mater. Chem. A Mater..

[24] Sun Y., Davis E. W. (2019). Facile Fabrication of Polydopamine Nanotubes for Combined
Chemo-Photothermal Therapy. J. Mater. Chem.
B.

[25] Xue J., Zheng W., Wang L., Jin Z. (2016). Scalable Fabrication
of Polydopamine Nanotubes Based on Curcumin Crystals. ACS Biomater. Sci. Eng..

[26] Bhardwaj R., Bharti A., Singh J. P., Chae K. H., Goyal N., Gautam S. (2018). Structural and Electronic
Investigation of ZnO Nanostructures
Synthesized under Different Environments. Heliyon.

[27] Luo H., Gu C., Zheng W., Dai F., Wang X., Zheng Z. (2015). Facile Synthesis
of Novel Size-Controlled Antibacterial Hybrid Spheres Using Silver
Nanoparticles Loaded with Poly-Dopamine Spheres. RSC Adv..

[28] Liu Y., Su C., Zu Y., Chen X., Sha J., Dai J. (2022). Ultrafast
Deposition of Polydopamine for High-Performance Fiber-Reinforced High-Temperature
Ceramic Composites. Sci. Rep..

[29] Bakry M., Ismail W., Abdelfatah M., El-Shaer A. (2024). Low-Cost Fabrication
Methods of ZnO Nanorods and Their Physical and Photoelectrochemical
Properties for Optoelectronic Applications. Sci. Rep..

[30] Liu R., Zhu Y., Jiang F., Fu Y., Zhang W., Zhang Y., Zhang G. (2021). Multifunctional Polydopamine
Particles as a Thermal Stability Modifier
to Prepare Antifouling Melt Blend Composite Membranes. ACS Omega.

[31] Antony
Lilly Grace M., Veerabhadra Rao K., Anuradha K., Judith Jayarani A., Arun kumar A., Rathika A. (2023). X-Ray Analysis and Size-Strain Plot
of Zinc Oxide Nanoparticles by Williamson-Hall. Mater. Today: Proc..

[32] Wang W., Li M., Huang X., Fang J., Peng F., Huang H. (2022). Structural
Evolution Mechanisms of Polydopamine/CdS and Photothermal Effect Boosted
Photocatalytic H2 Production Activity. Appl.
Surf. Sci..

[33] Wyatt P. J. (2021). Differential
Light Scattering and the Measurement of Molecules and Nanoparticles:
A Review. Anal. Chim. Acta X.

[34] Fan D., Zhu X., Zhai Q., Wang E., Dong S. (2016). Polydopamine
Nanotubes
as an Effective Fluorescent Quencher for Highly Sensitive and Selective
Detection of Biomolecules Assisted with Exonuclease III Amplification. Anal. Chem..

[35] Liebscher J., Mrówczyński R., Scheidt H. A., Filip C., Hădade N. D., Turcu R., Bende A., Beck S. (2013). Structure
of Polydopamine: A Never-Ending Story?. Langmuir.

[36] Sen D., Luhadiya N., Kundalwal S. I. (2025). Unravelling
Piezoelectric Properties
of Novel C3N and C3B Nanotubes: The First of Its Kind Atomistic Investigation. Diam. Relat. Mater..

[37] Srikanth, K. S. ; Wazeer, A. ; Mathiyalagan, P. ; Vidya, S. ; Rajput, K. ; Kushwaha, H. S. Piezoelectric Properties of ZnO. In Nanostructured Zinc Oxide; Elsevier, 2021; pp 717–736.

[38] Li F., Lin D., Chen Z., Cheng Z., Wang J., Li C., Xu Z., Huang Q., Liao X., Chen L.-Q., Shrout T. R., Zhang S. (2018). Ultrahigh Piezoelectricity in Ferroelectric Ceramics by Design. Nat. Mater..

[39] Kurita T., Terabayashi T., Kimura S., Numata K., Uji H. (2021). Construction
and Piezoelectric Properties of a Single-Peptide Nanotube Composed
of Cyclic β-Peptides with Helical Peptides on the Side Chains. Biomacromolecules.

[40] Ihlefeld, J. F. Fundamentals of Ferroelectric and Piezoelectric Properties. In Ferroelectricity in Doped Hafnium Oxide: Materials, Properties and Devices; Elsevier, 2019; pp 1–24.

[41] Kyriakides T. R., Raj A., Tseng T. H., Xiao H., Nguyen R., Mohammed F. S., Halder S., Xu M., Wu M. J., Bao S., Sheu W. C. (2021). Biocompatibility
of Nanomaterials and Their Immunological
Properties. Biomed. Mater.

[42] Ridolfo R., Tavakoli S., Junnuthula V., Williams D. S., Urtti A., van Hest J. C. M. (2021). Exploring the
Impact of Morphology on the Properties
of Biodegradable Nanoparticles and Their Diffusion in Complex Biological
Medium. Biomacromolecules.

[43] Deng J., Gao C. (2016). Recent Advances in
Interactions of Designed Nanoparticles and Cells
with Respect to Cellular Uptake, Intracellular Fate, Degradation and
Cytotoxicity. Nanotechnology.

[44] Pascua-Maestro R., Diez-Hermano S., Lillo C., Ganfornina M. D., Sanchez D. (2017). Protecting Cells by
Protecting Their Vulnerable Lysosomes:
Identification of a New Mechanism for Preserving Lysosomal Functional
Integrity upon Oxidative Stress. PLoS Genet..

[45] Dias V., Junn E., Mouradian M. M. (2013). The Role
of Oxidative Stress in Parkinson’s
Disease. J. Parkinson’s Dis..

[46] Manoharan S., Guillemin G. J., Abiramasundari R. S., Essa M. M., Akbar M., Akbar M. D. (2016). The Role
of Reactive Oxygen Species in the Pathogenesis
of Alzheimer’s Disease, Parkinson’s Disease, and Huntington’s
Disease: A Mini Review. Oxid. Med. Cell. Longev..

[47] Olmez I., Ozyurt H. (2012). Reactive Oxygen Species and Ischemic
Cerebrovascular
Disease. Neurochem. Int..

[48] Mittal M., Siddiqui M. R., Tran K., Reddy S. P., Malik A. B. (2014). Reactive
Oxygen Species in Inflammation and Tissue Injury. Antioxid. Redox Signal..

[49] Olivier C., Oliver L., Lalier L., Vallette F. M. (2021). Drug Resistance
in Glioblastoma: The Two Faces of Oxidative Stress. Front. Mol. Biosci..

[50] Zhang M., Ma Y., Ye X., Zhang N., Pan L., Wang B. (2023). TRP (Transient
Receptor Potential) Ion Channel Family: Structures, Biological Functions
and Therapeutic Interventions for Diseases. Signal Transduct. Target. Ther..

[51] Leach M. D., Cowen L. E. (2014). Membrane Fluidity and Temperature
Sensing Are Coupled
via Circuitry Comprised of Ole1, Rsp5, and Hsf1 in Candida Albicans. Eukaryot. Cell.

[52] Wu L., Lian W., Zhao L. (2021). Calcium Signaling in Cancer Progression
and Therapy. FEBS J..

[53] Brini M., Calì T., Ottolini D., Carafoli E. (2014). Neuronal Calcium Signaling:
Function and Dysfunction. Cell. Mol. Life Sci..

[54] Marino A., Battaglini M., De Pasquale D., Degl’Innocenti A., Ciofani G. (2018). Ultrasound-Activated
Piezoelectric Nanoparticles Inhibit
Proliferation of Breast Cancer Cells. Sci. Rep..

[55] Bargero A., Battaglini M., Curiale T., Carmignani A., Montorsi M., Labardi M., Pucci C., Marino A., Ciofani G. (2025). Ultrasound-Responsive
Polymeric Piezoelectric Nanoparticles
for Remote Activation and Neuronal Differentiation of Human Neural
Stem Cells. Small Sci..

[56] Pucci C., Marino A., Şen O. ¨., De Pasquale D., Bartolucci M., Iturrioz-Rodríguez N., di Leo N., de Vito G., Debellis D., Petretto A., Ciofani G. (2022). Ultrasound-Responsive
Nutlin-Loaded Nanoparticles for Combined Chemotherapy and Piezoelectric
Treatment of Glioblastoma Cells. Acta Biomater..

[57] Montorsi M., Pucci C., De Pasquale D., Marino A., Ceccarelli M. C., Mazzuferi M., Bartolucci M., Petretto A., Prato M., Debellis D., De Simoni G., Pugliese G., Labardi M., Ciofani G. (2024). Ultrasound-Activated
Piezoelectric Nanoparticles Trigger
Microglia Activity Against Glioblastoma Cells. Adv. Healthc. Mater..

[58] Marino A., Almici E., Migliorin S., Tapeinos C., Battaglini M., Cappello V., Marchetti M., de Vito G., Cicchi R., Pavone F. S., Ciofani G. (2019). Piezoelectric Barium Titanate Nanostimulators
for the Treatment of Glioblastoma Multiforme. J. Colloid Interface Sci..

[59] Nagar V., Singh T., Tiwari Y., Aseri V., Pandit P. P., Chopade R. L., Pandey K., Lodha P., Awasthi G. (2022). ZnO Nanoparticles:
Exposure, Toxicity Mechanism and Assessment. Mater. Today: Proc..

[60] Zhang S., Qin Y., Wang J., Yu Y., Wu L., Zhang T. (2023). Noninvasive
Electrical Stimulation Neuromodulation and Digital Brain Technology:
A Review. Biomedicines.

[61] Ciofani G., Ricotti L., Canale C., D’Alessandro D., Berrettini S., Mazzolai B., Mattoli V. (2013). Effects of Barium Titanate
Nanoparticles on Proliferation and Differentiation of Rat Mesenchymal
Stem Cells. Colloids Surf. B Biointerfaces.

[62] Fearnley C. J., Roderick H. L., Bootman M. D. (2011). Calcium
Signaling in Cardiac Myocytes. Cold Spring Harb.
Perspect. Biol..

[63] Chen X., Yang W., Zhang J., Zhang L., Shen H., Shi D. (2021). Alkalinity Triggered
the Degradation of Polydopamine Nanoparticles. Polym. Bull..

[64] Ma R., Chen Y. (2025). Progress on
Ultrasound-Responsive Piezoelectric Drug Delivery System
for Treatment of Neurodegenerative Diseases. J. Zhejiang Univ. Med. Sci..

[65] Della
Vecchia N. F., Avolio R., Alfè M., Errico M. E., Napolitano A., d’Ischia M. (2013). Building-Block
Diversity in Polydopamine Underpins a Multifunctional Eumelanin-Type
Platform Tunable Through a Quinone Control Point. Adv. Funct. Mater..

[66] Zhou Z. D., Yi L. X., Wang D. Q., Lim T. M., Tan E. K. (2023). Role of
Dopamine in the Pathophysiology of Parkinson’s Disease. Transl. Neurodegener..

[67] Antonini A., Odin P. (2009). Pros and Cons of Apomorphine
and L-Dopa Continuous Infusion in Advanced
Parkinson’s Disease. Parkinsonism Relat.
Disord..

[68] Mishra A., Singh S., Tiwari V., Parul, Shukla S. (2019). Dopamine D1
Receptor
Activation Improves Adult Hippocampal Neurogenesis and Exerts Anxiolytic
and Antidepressant-like Effect via Activation of Wnt/β-Catenin
Pathways in Rat Model of Parkinson’s Disease. Neurochem. Int..

[69] Speranza L., di Porzio U., Viggiano D., de Donato A., Volpicelli F. (2021). Dopamine:
The Neuromodulator of Long-Term Synaptic
Plasticity, Reward and Movement Control. Cells.

[70] Salgado-Pineda P., Delaveau P., Blin O., Nieoullon A. (2005). Dopaminergic
Contribution to the Regulation of Emotional Perception. Clin. Neuropharmacol..

[71] Tapeinos C., Battaglini M., Ciofani G. (2017). Advances in the Design of Solid Lipid
Nanoparticles and Nanostructured Lipid Carriers for Targeting Brain
Diseases. J. Controlled Release.

[72] Martinelli C., Pucci C., Battaglini M., Marino A., Ciofani G. (2020). Antioxidants
and Nanotechnology: Promises and Limits of Potentially Disruptive
Approaches in the Treatment of Central Nervous System Diseases. Adv. Healthc. Mater..

[73] Liu R., Shin K. H., Zhu Y., Liu Q., Ji B., Sun G., Li Z., De Silva D., Stewart A., Lorenzoni M., Ludtke I., Williams O. A., Ming W., Divitini G., Sohn J. I., Hou B. (2025). Modulating D _33_ Coefficients
Through In Situ AgF and Ag _2_ O Growth in PVDF Composites
for High-Performance Piezoelectric Nanogenerators. Adv. Mater. Technol..

[74] Marino A., Camponovo A., Degl’Innocenti A., Bartolucci M., Tapeinos C., Martinelli C., De Pasquale D., Santoro F., Mollo V., Arai S., Suzuki M., Harada Y., Petretto A., Ciofani G. (2019). Multifunctional Temozolomide-Loaded
Lipid Superparamagnetic Nanovectors: Dual Targeting and Disintegration
of Glioblastoma Spheroids by Synergic Chemotherapy and Hyperthermia
Treatment. Nanoscale.

[75] Marino A., Arai S., Hou Y., Sinibaldi E., Pellegrino M., Chang Y.-T., Mazzolai B., Mattoli V., Suzuki M., Ciofani G. (2015). Piezoelectric Nanoparticle-Assisted
Wireless Neuronal Stimulation. ACS Nano.

[76] Rajabi A. H., Jaffe M., Arinzeh T. L. (2015). Piezoelectric
Materials for Tissue
Regeneration: A Review. Acta Biomater..

[77] Tandon B., Blaker J. J., Cartmell S. H. (2018). Piezoelectric
Materials as Stimulatory
Biomedical Materials and Scaffolds for Bone Repair. Acta Biomater..

[78] Chernozem R. V., Guselnikova O., Surmeneva M. A., Postnikov P. S., Abalymov A. A., Parakhonskiy B. V., De Roo N., Depla D., Skirtach A. G., Surmenev R. A. (2020). Diazonium
Chemistry Surface Treatment
of Piezoelectric Polyhydroxybutyrate Scaffolds for Enhanced Osteoblastic
Cell Growth. Appl. Mater. Today.

[79] Chorsi M. T., Curry E. J., Chorsi H. T., Das R., Baroody J., Purohit P. K., Ilies H., Nguyen T. D. (2019). Piezoelectric
Biomaterials
for Sensors and Actuators. Adv. Mater..

[80] Guy I. L., Muensit S., Goldys E. M. (1999). Extensional Piezoelectric
Coefficients
of Gallium Nitride and Aluminum Nitride. Appl.
Phys. Lett..

[81] Zhao G., Huang B., Zhang J., Wang A., Ren K., Wang Z. L. (2017). Electrospun Poly­(l-Lactic
Acid) Nanofibers for Nanogenerator
and Diagnostic Sensor Applications. Macromol.
Mater. Eng..

[82] Mishra S., Unnikrishnan L., Nayak S. K., Mohanty S. (2019). Advances in Piezoelectric
Polymer Composites for Energy Harvesting Applications: A Systematic
Review. Macromol. Mater. Eng..

[83] Liu S., Cui Z., Fu P., Liu M., Zhang L., Li Z., Zhao Q. (2014). Ferroelectric Behavior and Polarization Mechanism in
Odd-odd Polyamide
11,11. J. Polym. Sci. B Polym. Phys..

[84] Akiyama M., Kamohara T., Kano K., Teshigahara A., Takeuchi Y., Kawahara N. (2009). Enhancement of Piezoelectric
Response
in Scandium Aluminum Nitride Alloy Thin Films Prepared by Dual Reactive
Cosputtering. Adv. Mater..

[85] Guo S., Duan X., Xie M., Aw K. C., Xue Q. (2020). Composites,
Fabrication and Application of Polyvinylidene Fluoride for Flexible
Electromechanical Devices: A Review. Micromachines
(Basel)..

[86] Abu
Ali T., Pilz J., Schäffner P., Kratzer M., Teichert C., Stadlober B., Coclite A. M. (2020). Piezoelectric Properties of Zinc
Oxide Thin Films Grown by Plasma-Enhanced Atomic Layer Deposition. physica status solidi (a).

[87] Costa J., Peixoto T., Ferreira A., Vaz F., Lopes M. A. (2019). Development
and Characterization of ZnO Piezoelectric Thin Films on Polymeric
Substrates for Tissue Repair. J. Biomed. Mater.
Res., Part A.

[88] Wang, Y. ; Jiang, Y. Dielectric and Piezoelectric Anisotropy of Lithium Niobate and Lithium Tantalate Single Crystals. In 2009 18th IEEE International Symposium on the Applications of Ferroelectrics; IEEE, 2009, pp 1–4.

[89] Sharma T., Je S.-S., Gill B., Zhang J. X. J. (2012). Patterning
Piezoelectric
Thin Film PVDF-TrFE Based Pressure Sensor for Catheter Application. Sens. Actuators A Phys..

[90] Zhou Z., Li J., Xia W., Zhu X., Sun T., Cao C., Zhang L. (2020). Enhanced Piezoelectric
and Acoustic Performances of Poly­(Vinylidene
Fluoride-Trifluoroethylene) Films for Hydroacoustic Applications. Phys. Chem. Chem. Phys..

[91] Lian L., Sottos N. R. (2000). Effects of Thickness on the Piezoelectric and Dielectric
Properties of Lead Zirconate Titanate Thin Films. J. Appl. Phys..

[92] Li P., Zhai J., Shen B., Zhang S., Li X., Zhu F., Zhang X. (2018). Ultrahigh Piezoelectric Properties in Textured (K,Na)­NbO _3_ -Based Lead-Free Ceramics. Adv. Mater..

[93] Gao X., Cheng Z., Chen Z., Liu Y., Meng X., Zhang X., Wang J., Guo Q., Li B., Sun H., Gu Q., Hao H., Shen Q., Wu J., Liao X., Ringer S. P., Liu H., Zhang L., Chen W., Li F., Zhang S. (2021). The Mechanism for the
Enhanced Piezoelectricity in Multi-Elements Doped (K,Na)­NbO3 Ceramics. Nat. Commun..

[94] Acosta M., Novak N., Rojas V., Patel S., Vaish R., Koruza J., Rossetti G. A., Rödel J. (2017). BaTiO3-Based
Piezoelectrics: Fundamentals, Current Status, and Perspectives. Appl. Phys. Rev..

