# Supplementary material for: Polydopamine Nanotubes: Multifunctional Smart Nanotransducers for Cellular Activity Modulation
Source: ACS Nano. 2026 Aug 7;20(32):22616–34. doi: 10.1021/acsnano.6c05512 (PMC13492472; doi:10.1021/acsnano.6c05512)
Supplement: Supplementary file 1 [file nn6c05512_si_001.pdf]

Polydopamine Nanotubes: Multifunctional Smart Nanotransducers for Cellular Activity Modulation

Matteo Battaglini[1*], Attilio Marino[1], Tommaso Curiale[1], Alessio Carmignani[1], Margherita Bernardeschi[1], Melis Emanet[1], Maria Cristina Ceccarelli[1,2], Federico Catalano[3], Filippo Drago[4], Sergio Marras[5], Giammarino Pugliese[4], Riccardo Carzino[5], Bruno Torre[6], Gianni Ciofani[1*]

[1] Istituto Italiano di Tecnologia, Smart Bio-Interfaces, Viale Rinaldo Piaggio 34, 56025 Pontedera, Italy

[2] Scuola Superiore Sant'Anna, The Biorobotics Institute, Viale Rinaldo Piaggio 34, 56025 Pontedera, Italy

[3] Istituto Italiano di Tecnologia, Electron Microscopy Facility, Via Morego 30, 16163 Genova, Italy

[4] Istituto Italiano di Tecnologia, Nanochemistry, Via Morego 30, 16163 Genova, Italy

[5] Istituto Italiano di Tecnologia, Materials Characterization Facility, Via Morego 30, 16163 Genova, Italy

[6] Istituto Nazionale di Ricerca Metrologica INRiM, Advanced Materials and Life Science Division, Strada delle Cacce 91, 10135 Torino, Italy

*Corresponding Authors: matteo.battaglini@iit.it; gianni.ciofani@iit.it

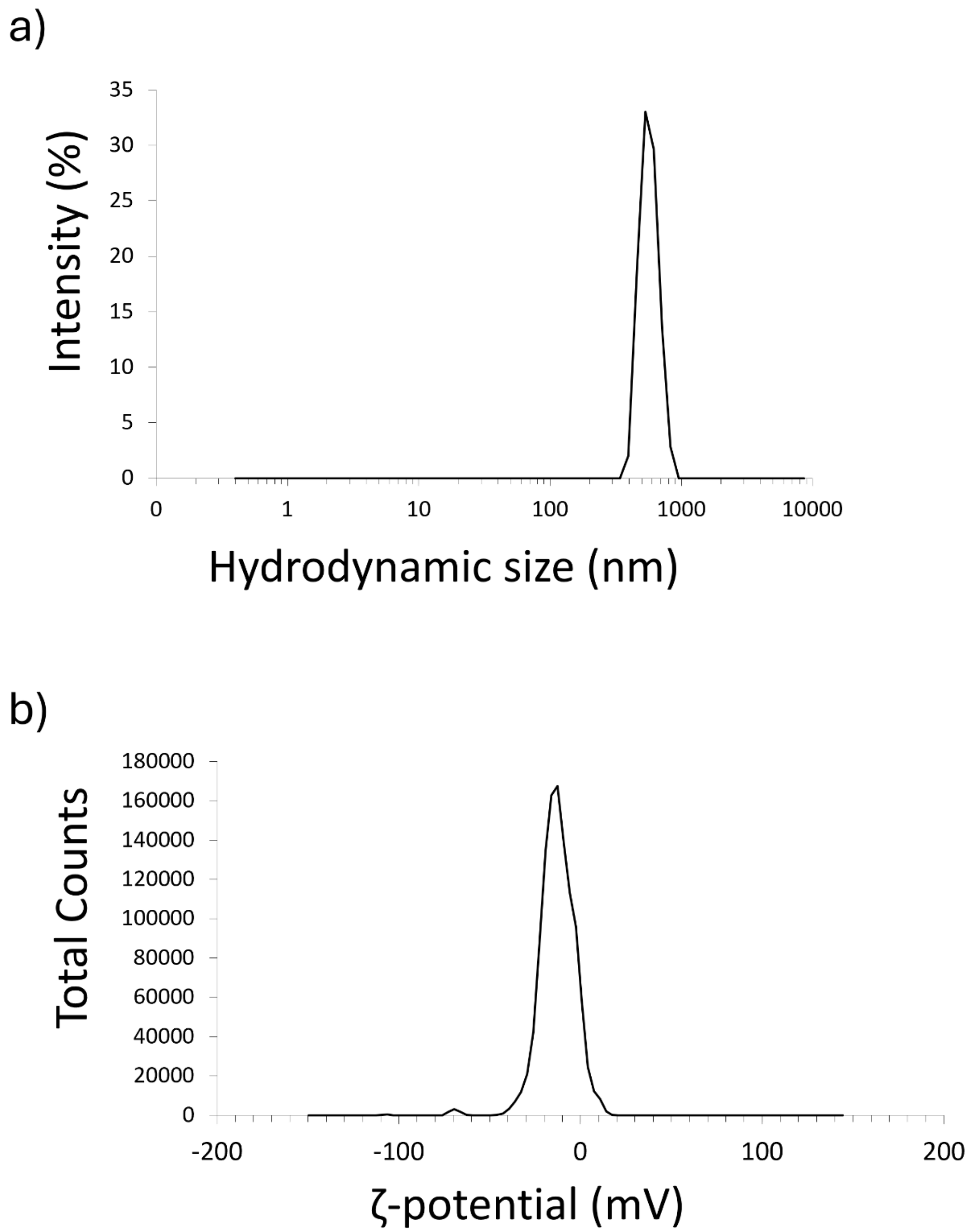

Figure S1 a) DLS and b) ζ-potential analysis of PDA-NTs.

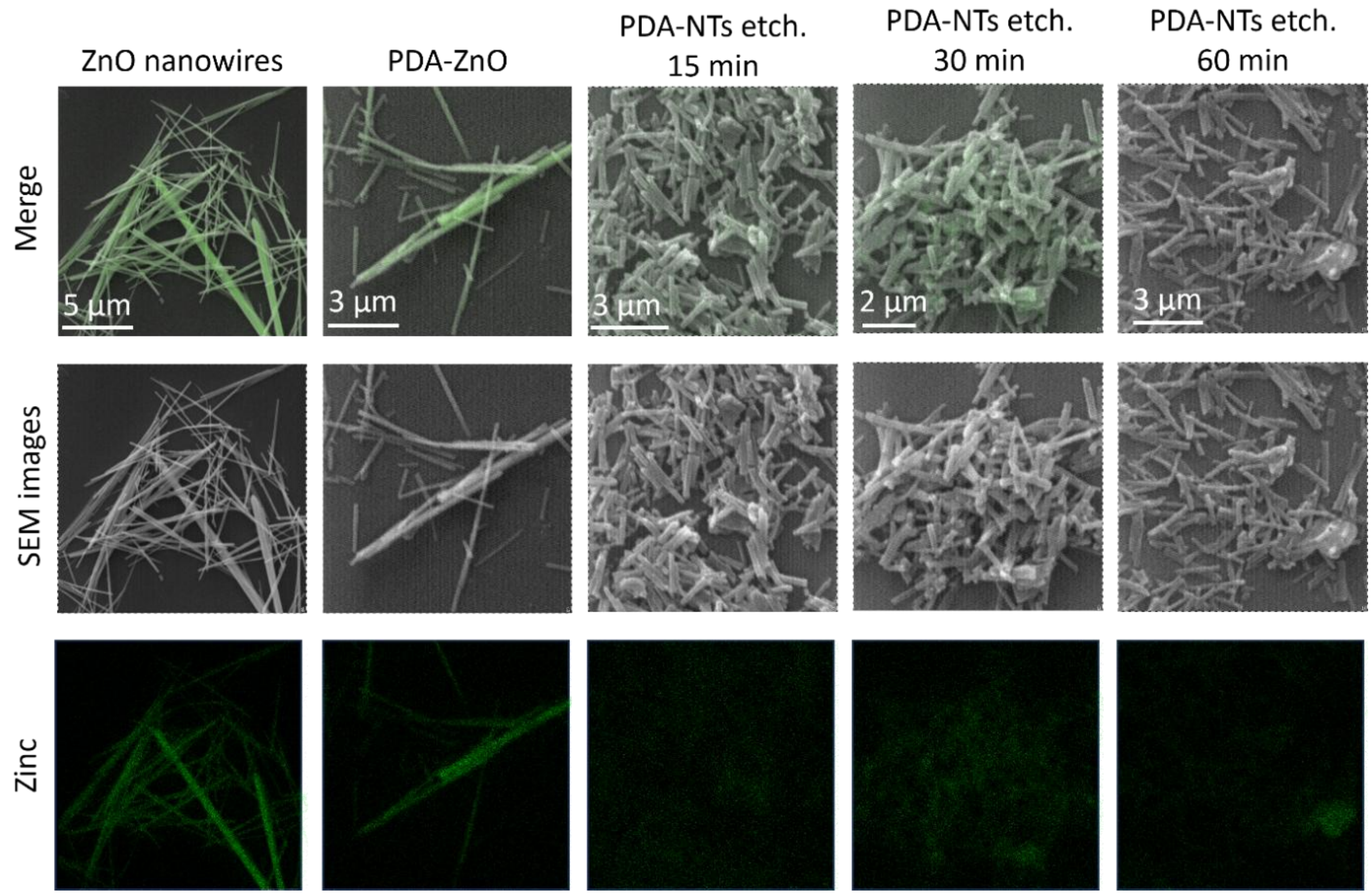

Figure S2. Representative SEM images and corresponding EDS elemental mapping of Zn for ZnO nanowires, PDA-ZnO, and PDA-NTs after different etching times (15, 30, and 60 min). The merged images combine SEM micrographs with Zn elemental distribution maps.

a)

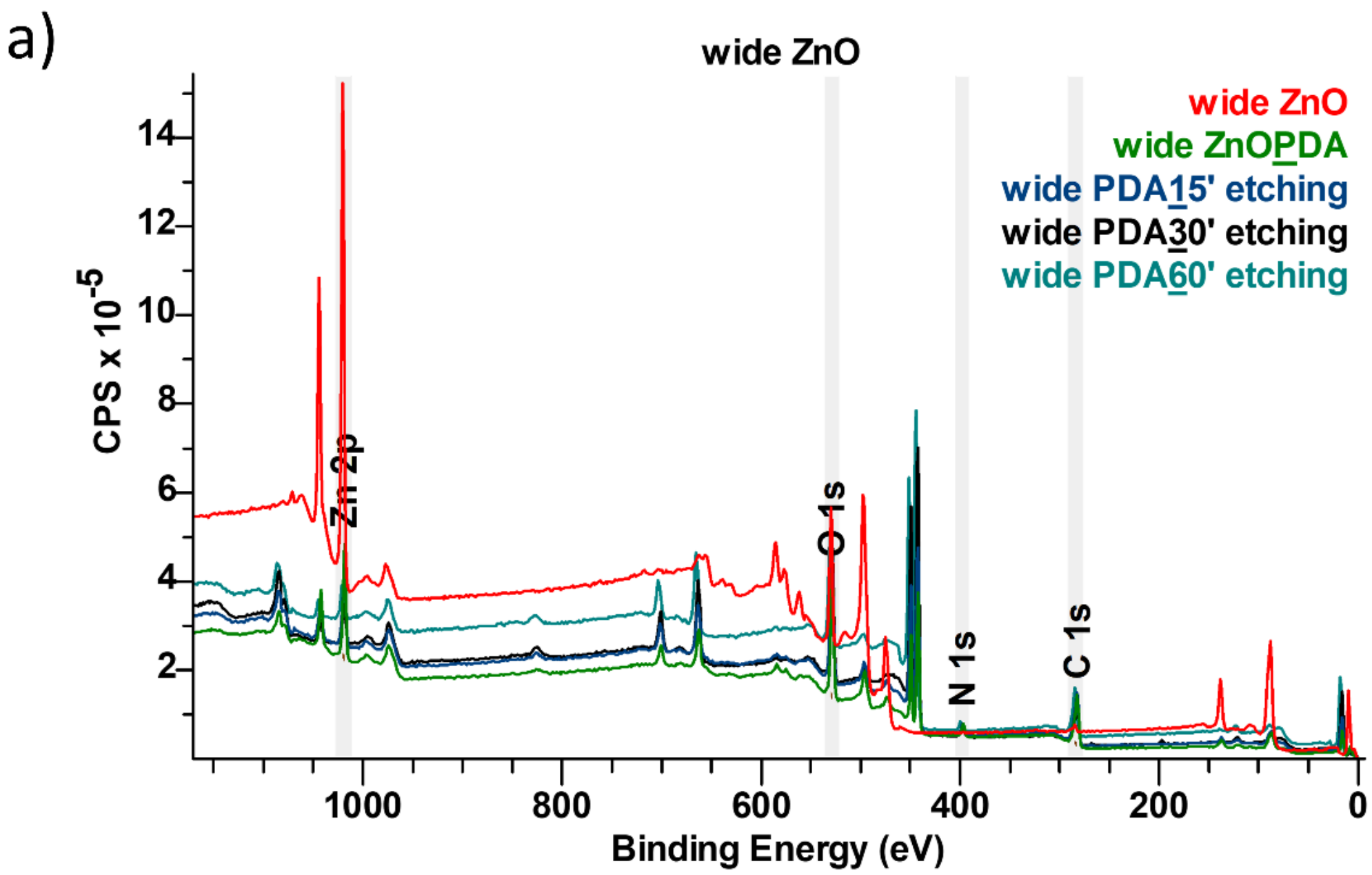

b)

	C (At%)	O (At%)	N (At%)	Zn (At%)
ZnO Nanowires	8.9	52.7	-	38.4
PDA-ZnO	58.2	31.3	5.2	5.3
PDA-NTs etch. 15 min	56.5	33.9	5.5	4.1
PDA-NTs etch. 30 min	52.6	40.4	4.8	2.2
PDA-NTs etch. 60 min	54.5	38.5	5.0	2.0

c)

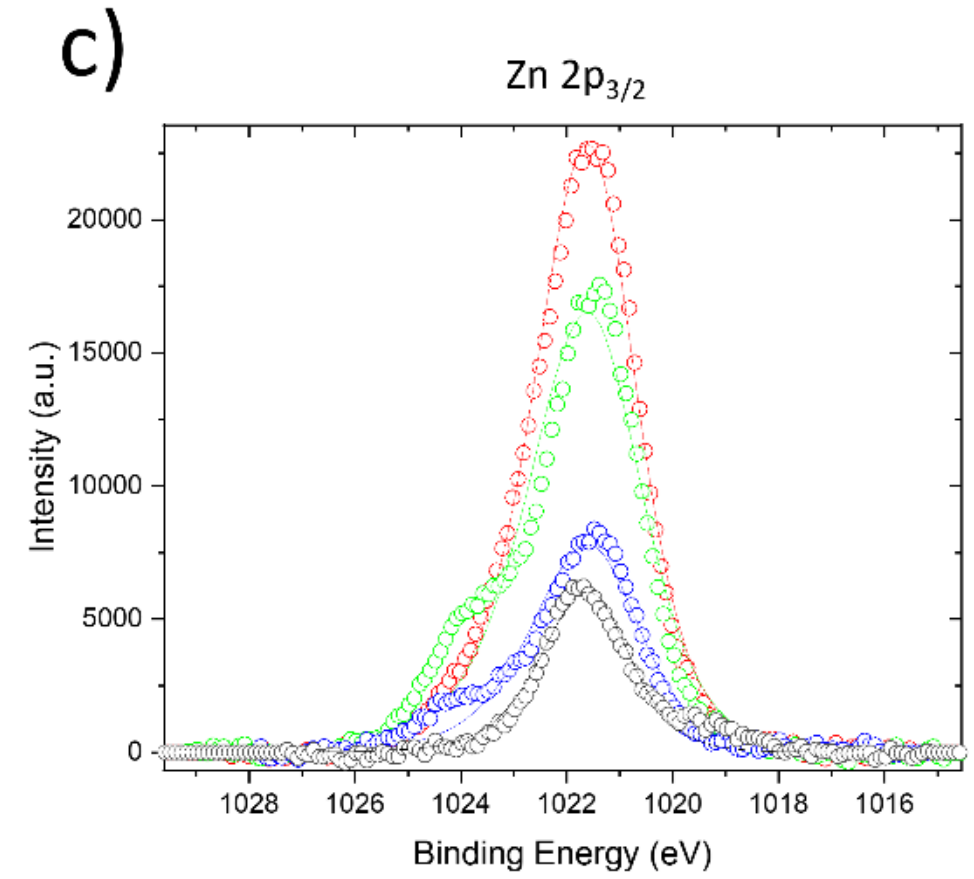

PDA-ZnO — PDA-NTs etch. 15 min

PDA-NTs etch. 30 min — PDA-NTs etch. 60 min

Figure S3. a) XPS survey spectra of pristine ZnO nanowires, PDA-coated ZnO (PDA–ZnO), and PDA nanotubes (PDA-NTs) obtained after different etching times (15, 30, and 60 min). b) Surface elemental composition (At%) derived from XPS analysis for ZnO nanowires, PDA–ZnO, and PDA-NTs at different etching times. c) High-resolution XPS spectra of the Zn $2p_{3/2}$ region for PDA–ZnO and PDA-NTs after different etching times.

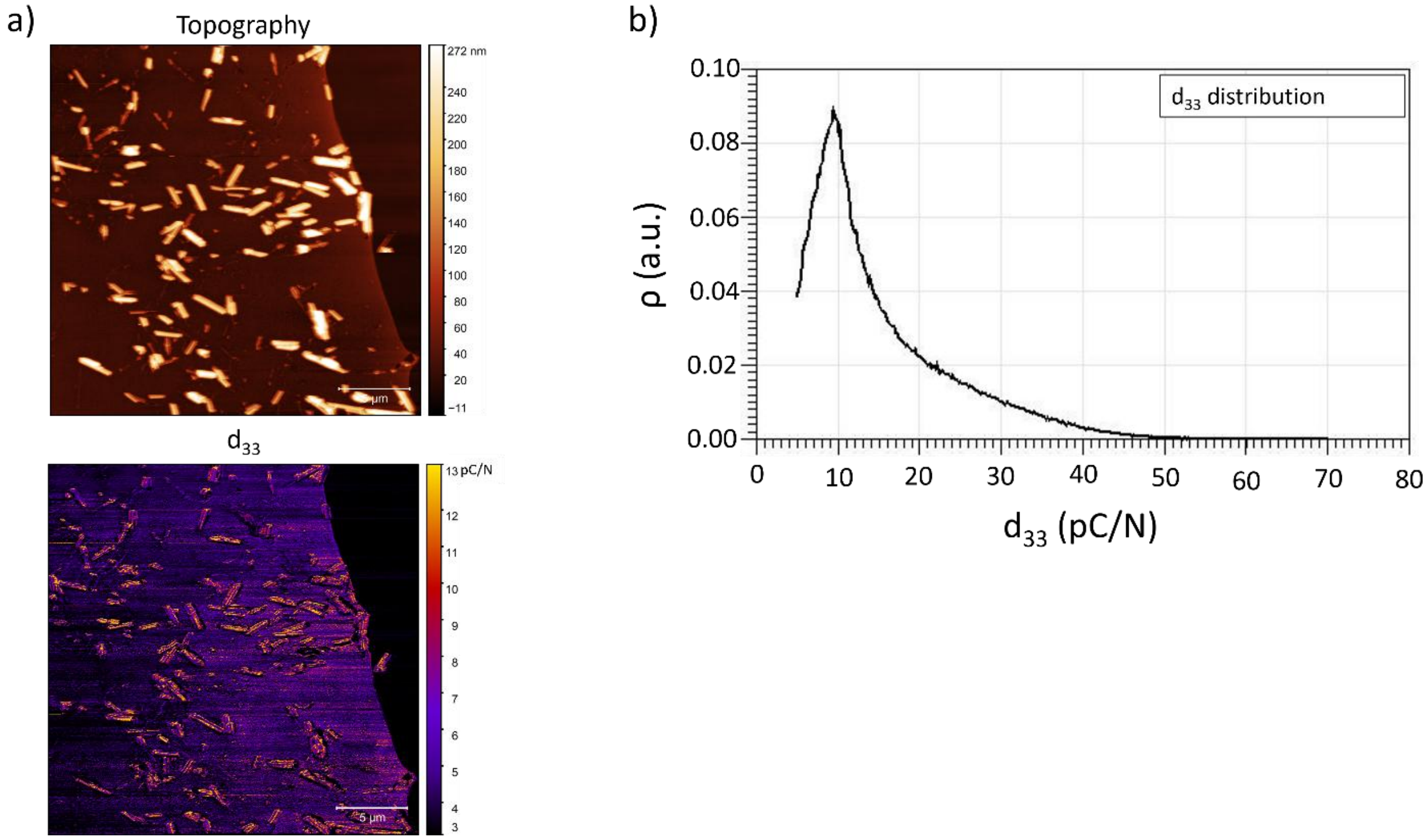

Figure S4. a) AFM topography image (top) and corresponding PFM d_{33} map (bottom) of PDA-NTs. b) Distribution of the d_{33} piezoelectric coefficient measured for PDA-NTs, showing the statistical spread of the electromechanical response obtained from PFM analysis.

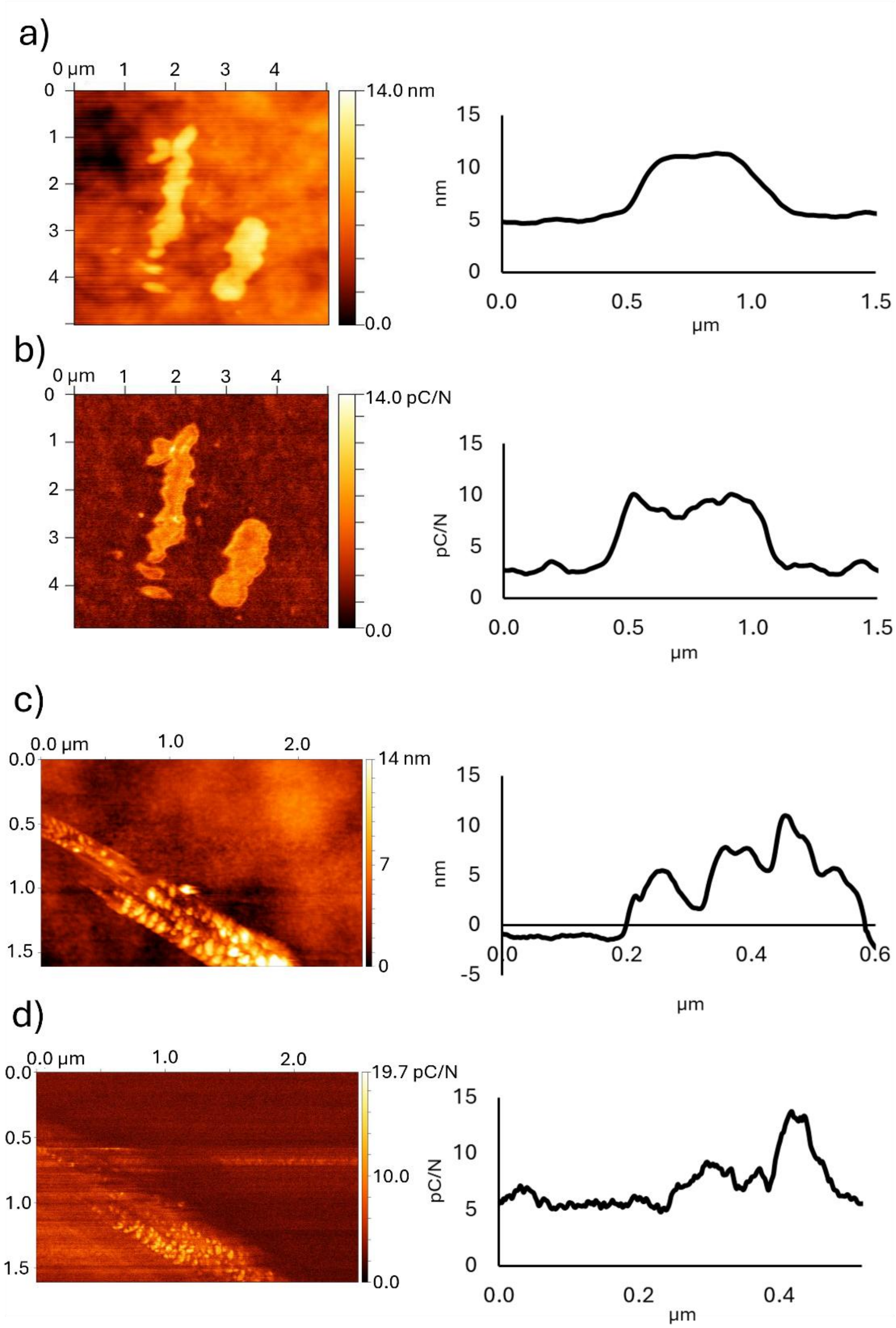

Figure S5. AFM (a, c) and PFM (b, d) characterization of ZnO nanowires (a, b) and PDA-ZnO (c, d). On the left representative images, on the right quantitative analysis.

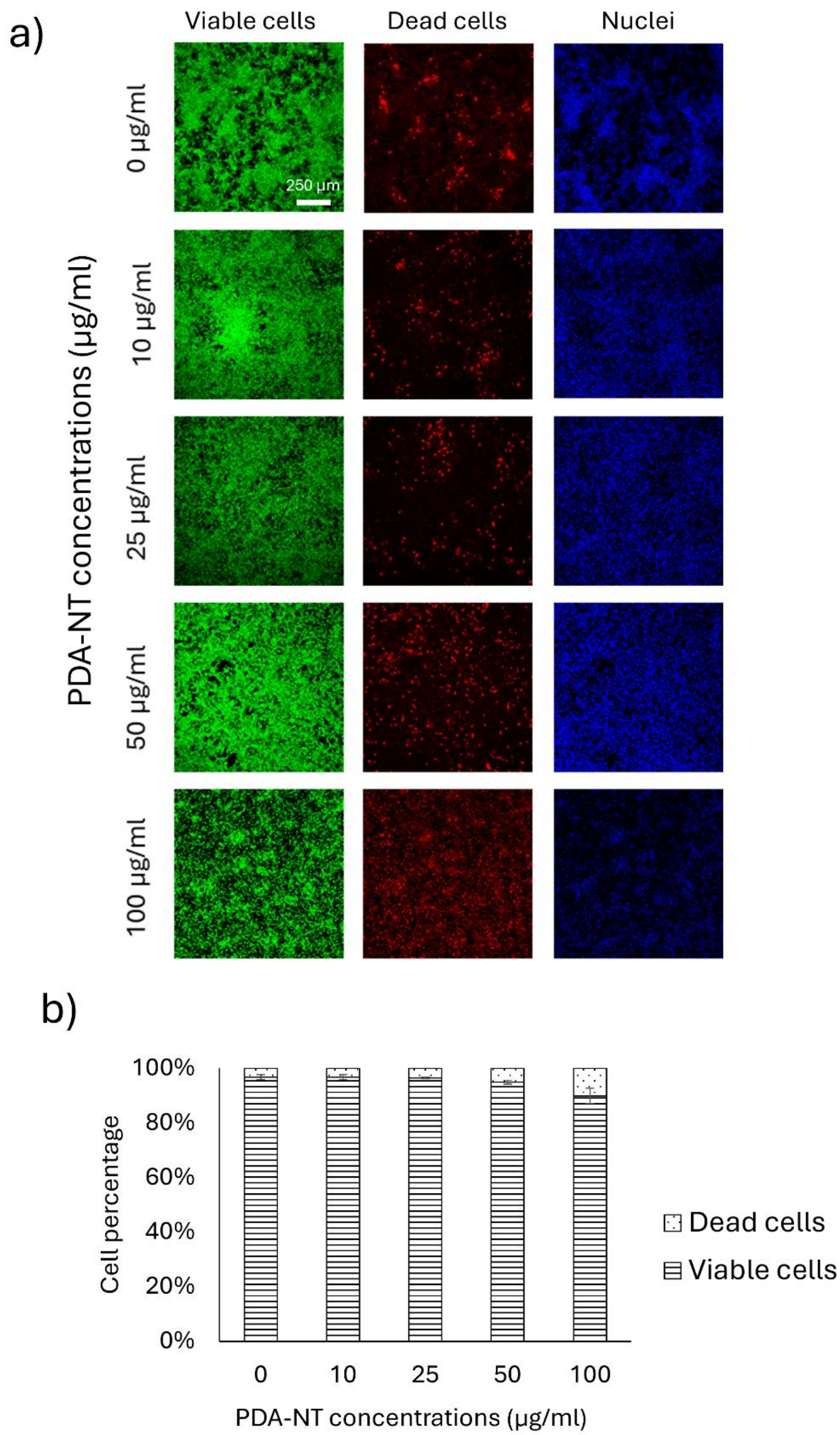

Figure S6. a) Representative confocal images and b) quantitative evaluation the Live/Dead assay.

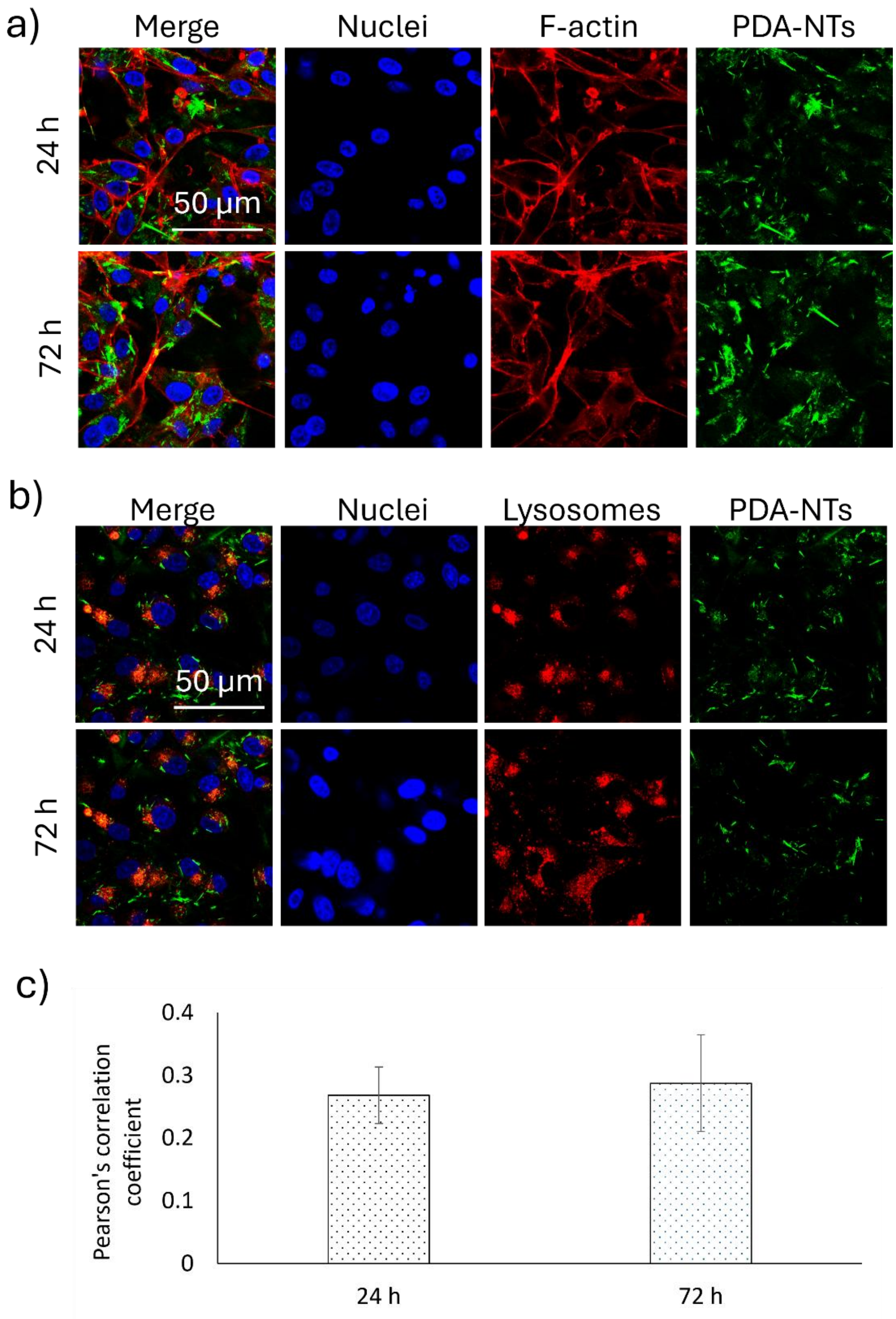

Figure S7. Representative confocal images showing a) the internalization of PDA-NTs in U87 MG cells and b) their intracellular localization with respect to lysosomes. c) Quantitative analysis of the Pearson's correlation coefficient describing lysosomal co-localization.

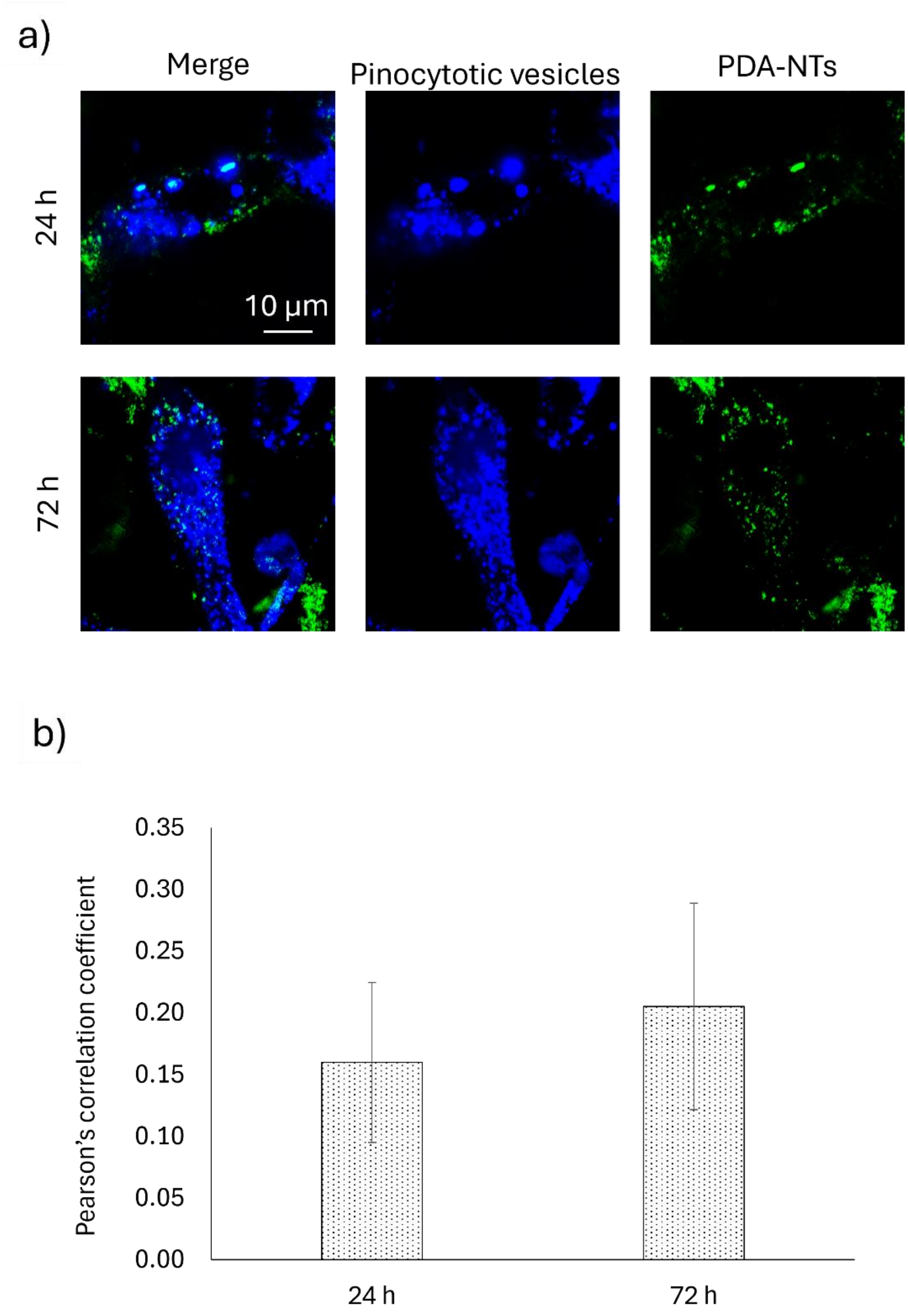

Figure S8. a) Confocal fluorescence images showing PDA-NTs and pinocytotic vesicles (blue), along with merged images highlighting nanoparticle spatial distribution and overlap within cells. b) Corresponding colocalization analysis quantifying the degree of overlap between PDA-NTs and pinocytotic vesicles, presented as mean ± standard deviation.

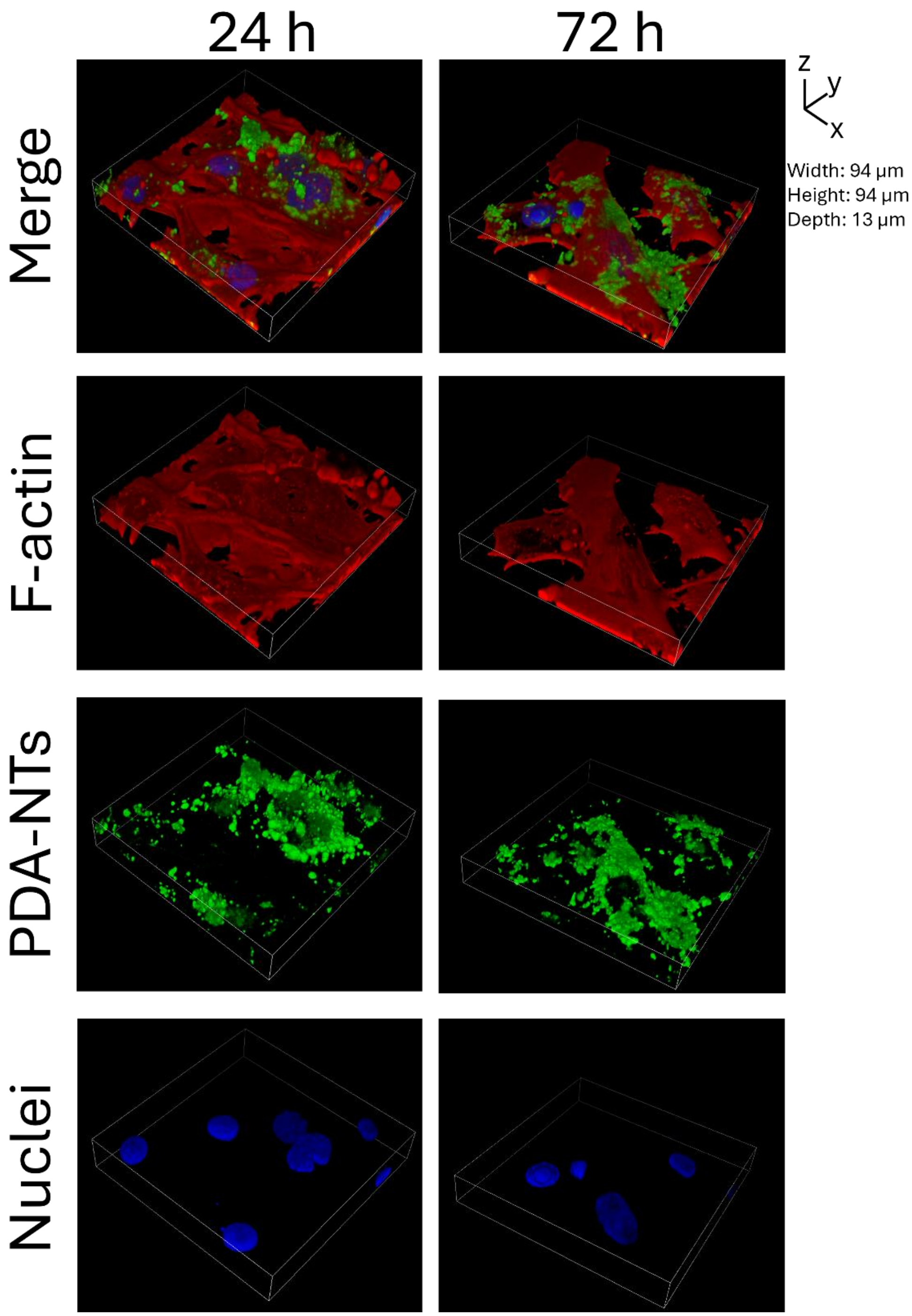

Figure S9. Representative 3D renderings of confocal microscopy images illustrating the internalization of PDA-NTs within cells. Cell f-actin shown in red and nuclei in blue, with separated channels and merged views highlighting the intracellular distribution of PDA-NTs.

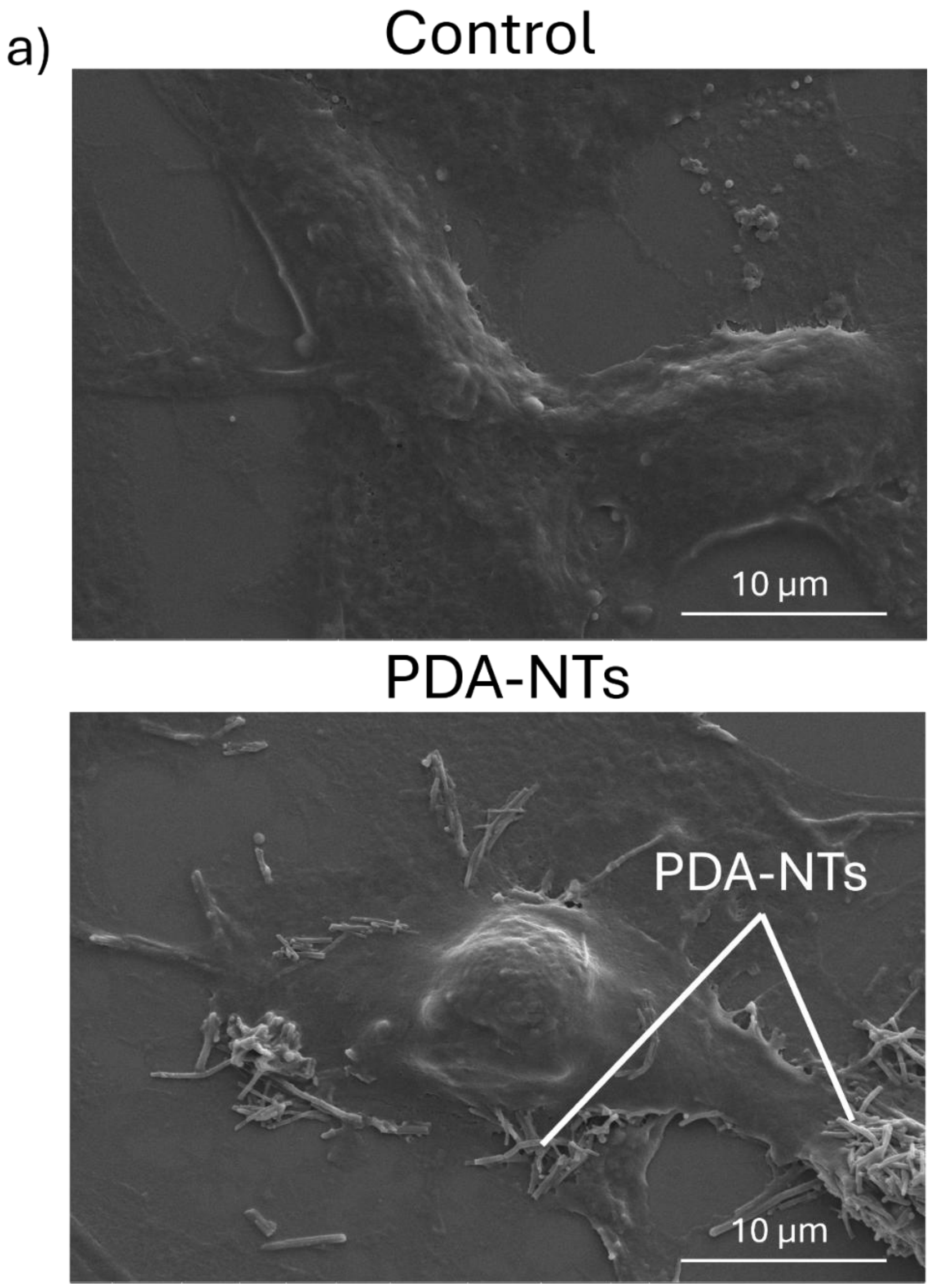

Figure S10. SEM images showing U87 MG cells a) non-treated and b) PDA-NTs-treated.

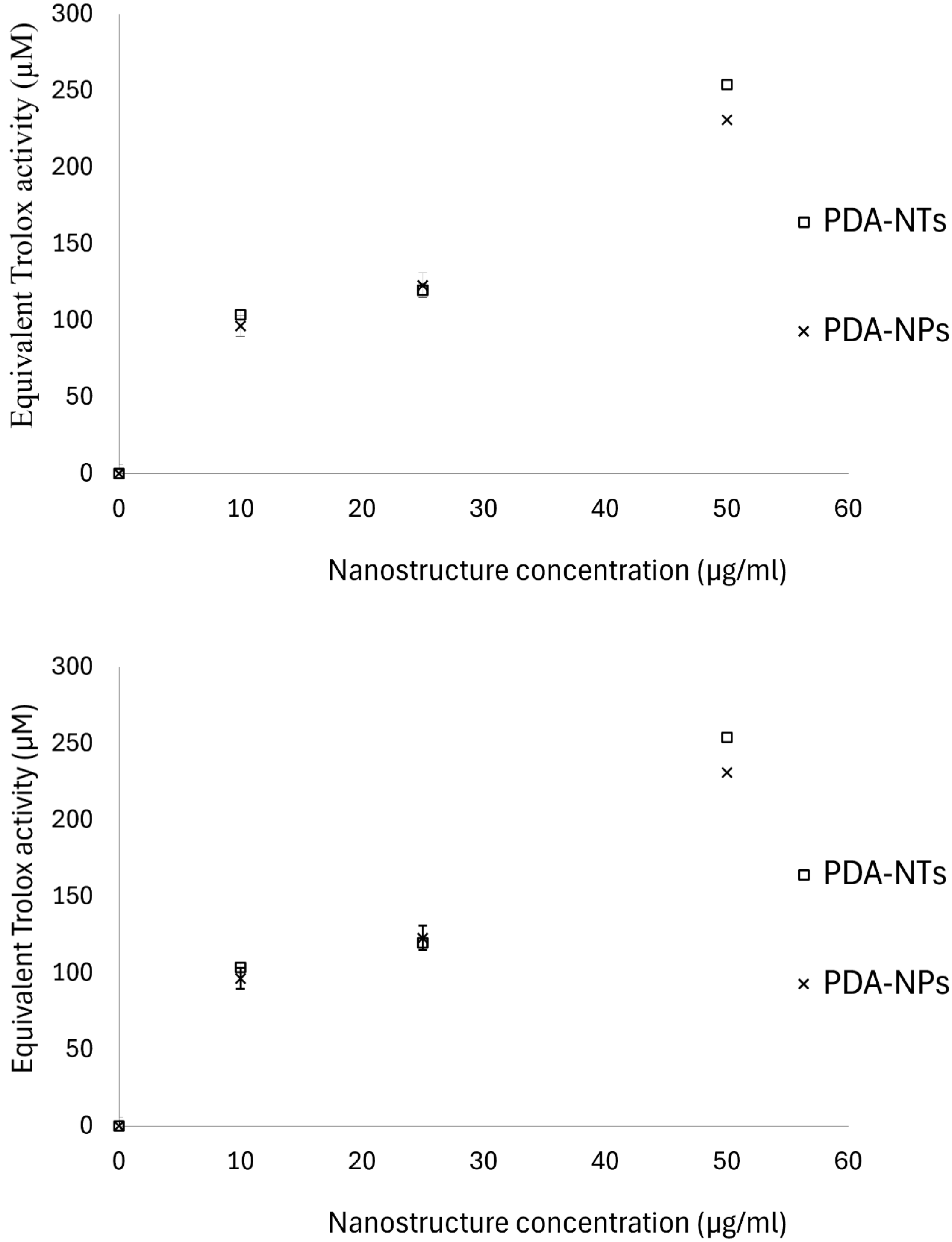

Figure S11. Antioxidant capacity of PDA-NTs and PDA-NPs at various concentrations, expressed as Trolox equivalent.

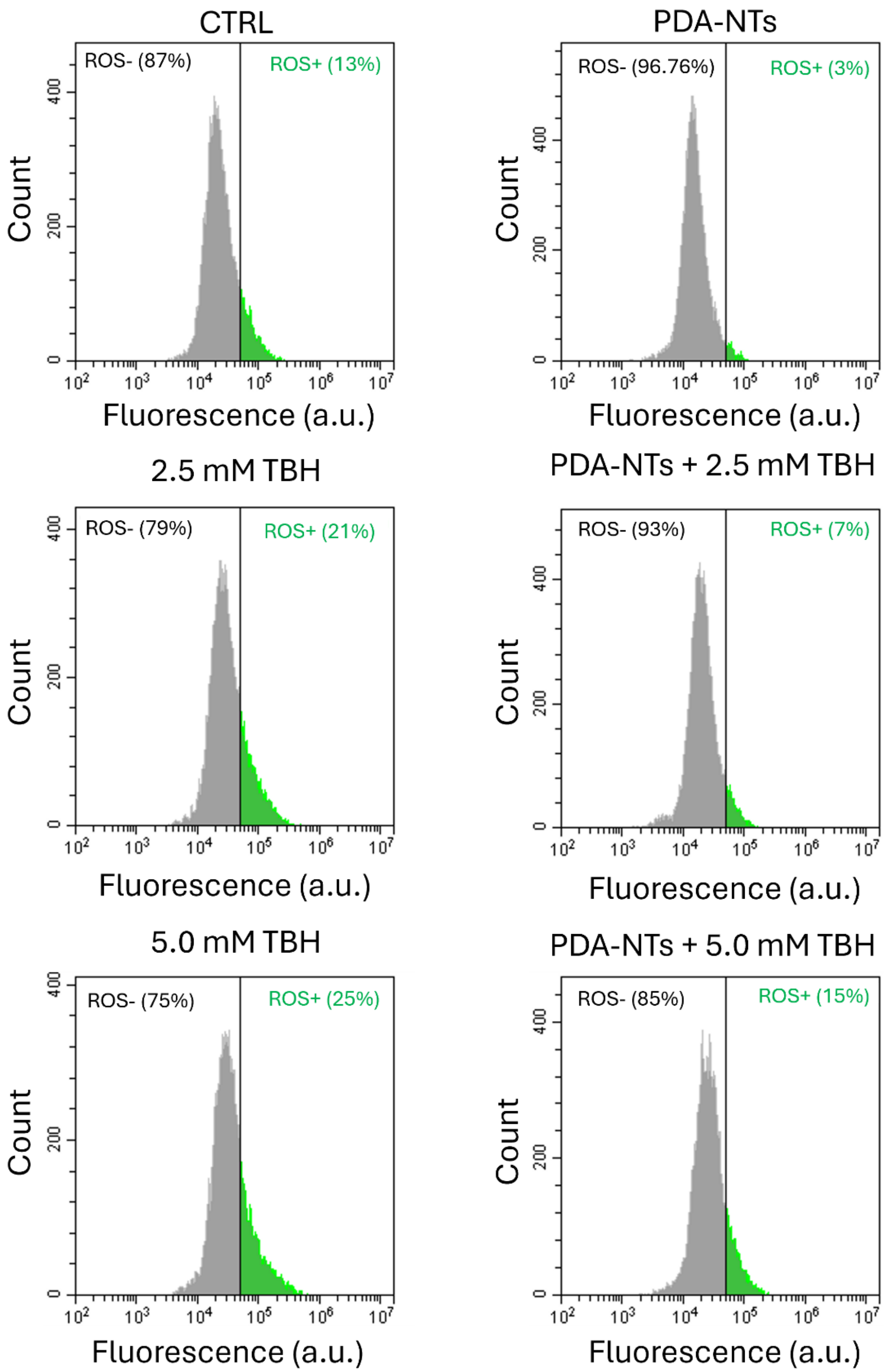

Figure S12. Representative flow cytometry histograms showing cytosolic ROS analysis in U87 MG cells for different experimental conditions.

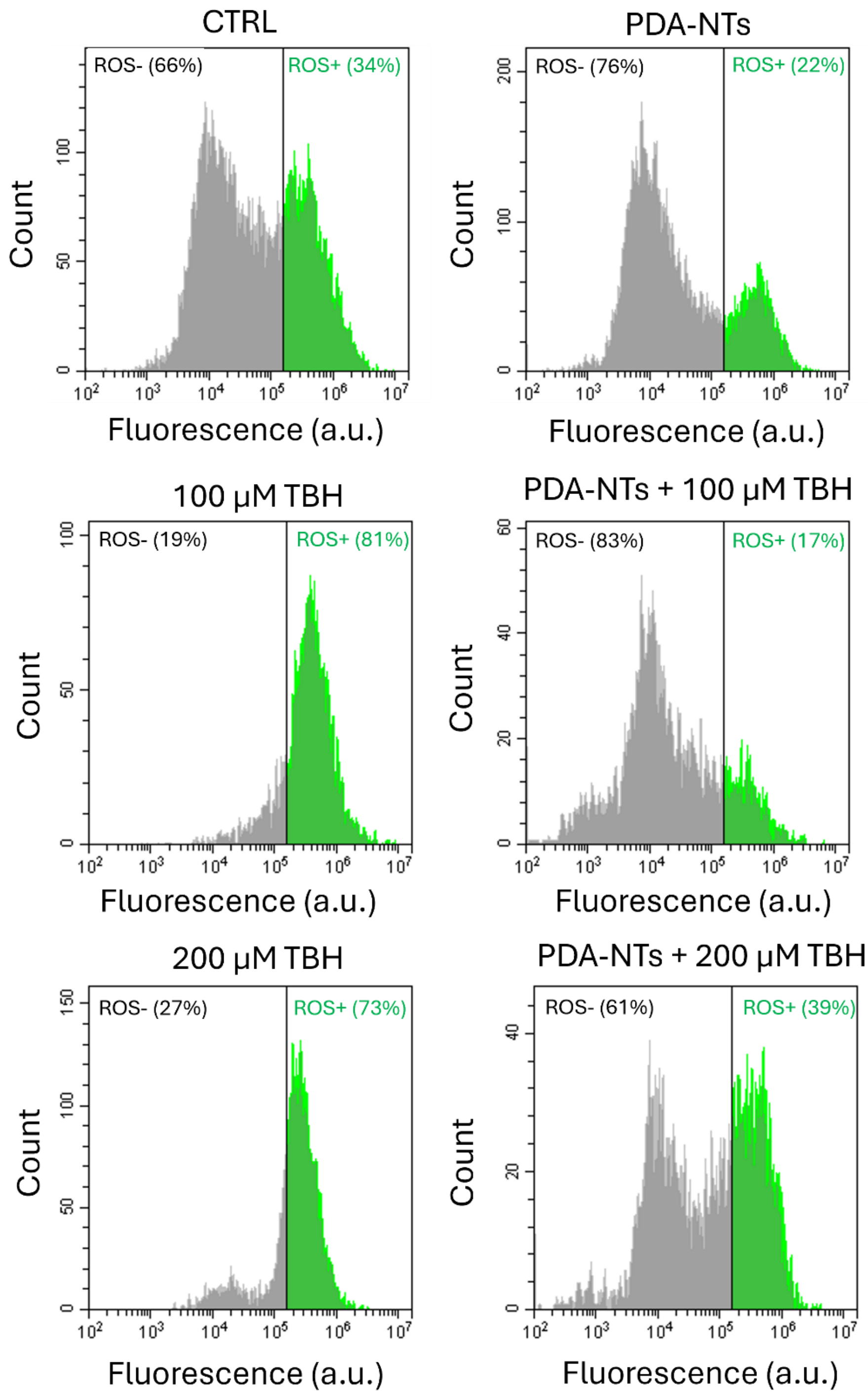

Figure S13. Representative flow cytometry histograms showing mitochondrial ROS analysis in U87 MG cells for different experimental conditions.

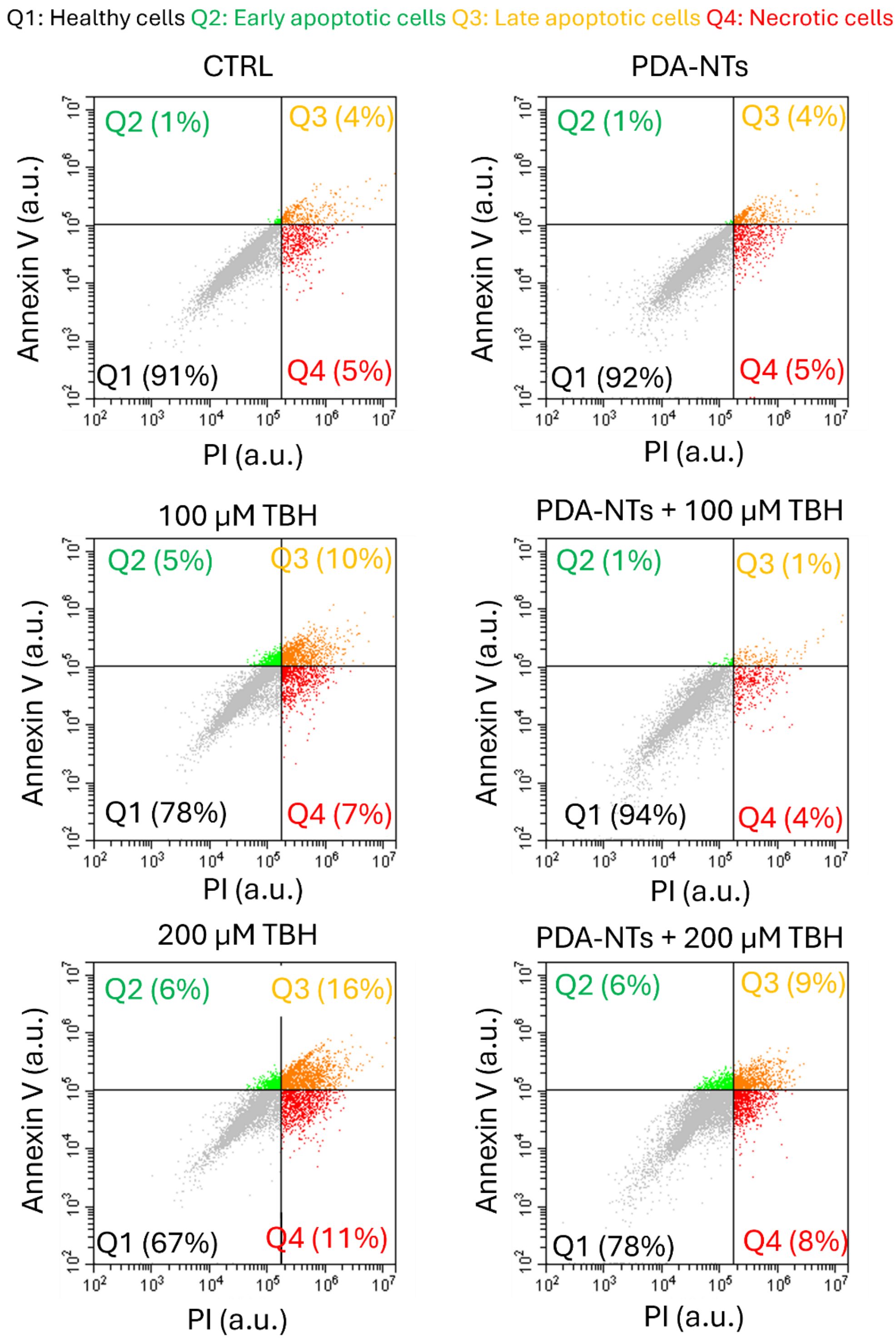

Figure S14 Representative dot plots of flow cytometry analysis showing the apoptosis/necrosis levels of U87 MG cells for different experimental conditions.

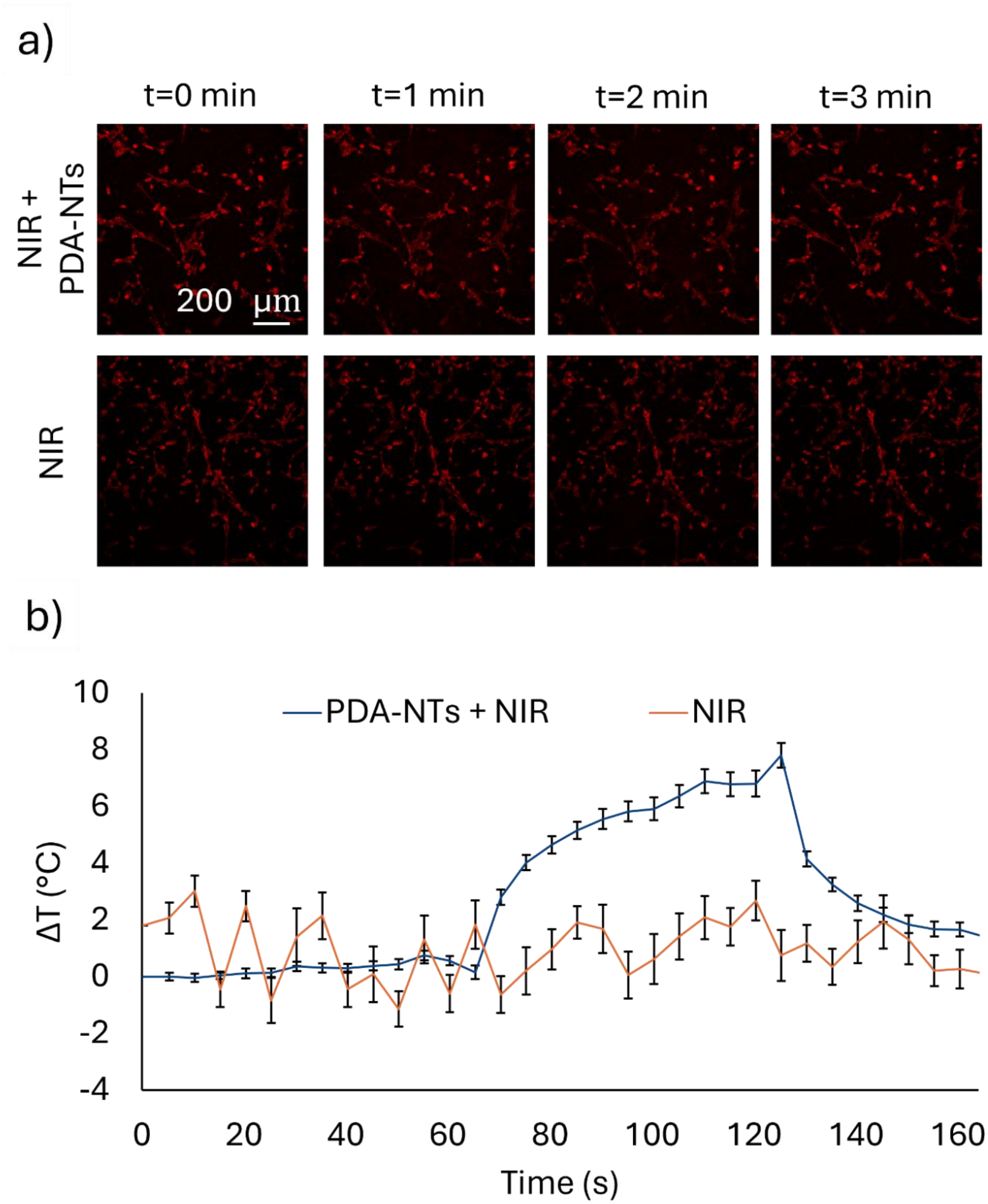

Figure S15. a) Representative confocal fluorescence images showing changes in cellular fluorescence intensity upon NIR irradiation in the presence and absence of PDA-NTs (scale bar: 200 µm), indicative of temperature increment. b) Corresponding temperature–time profiles, demonstrating heating in samples containing PDA-NTs with respect to controls.

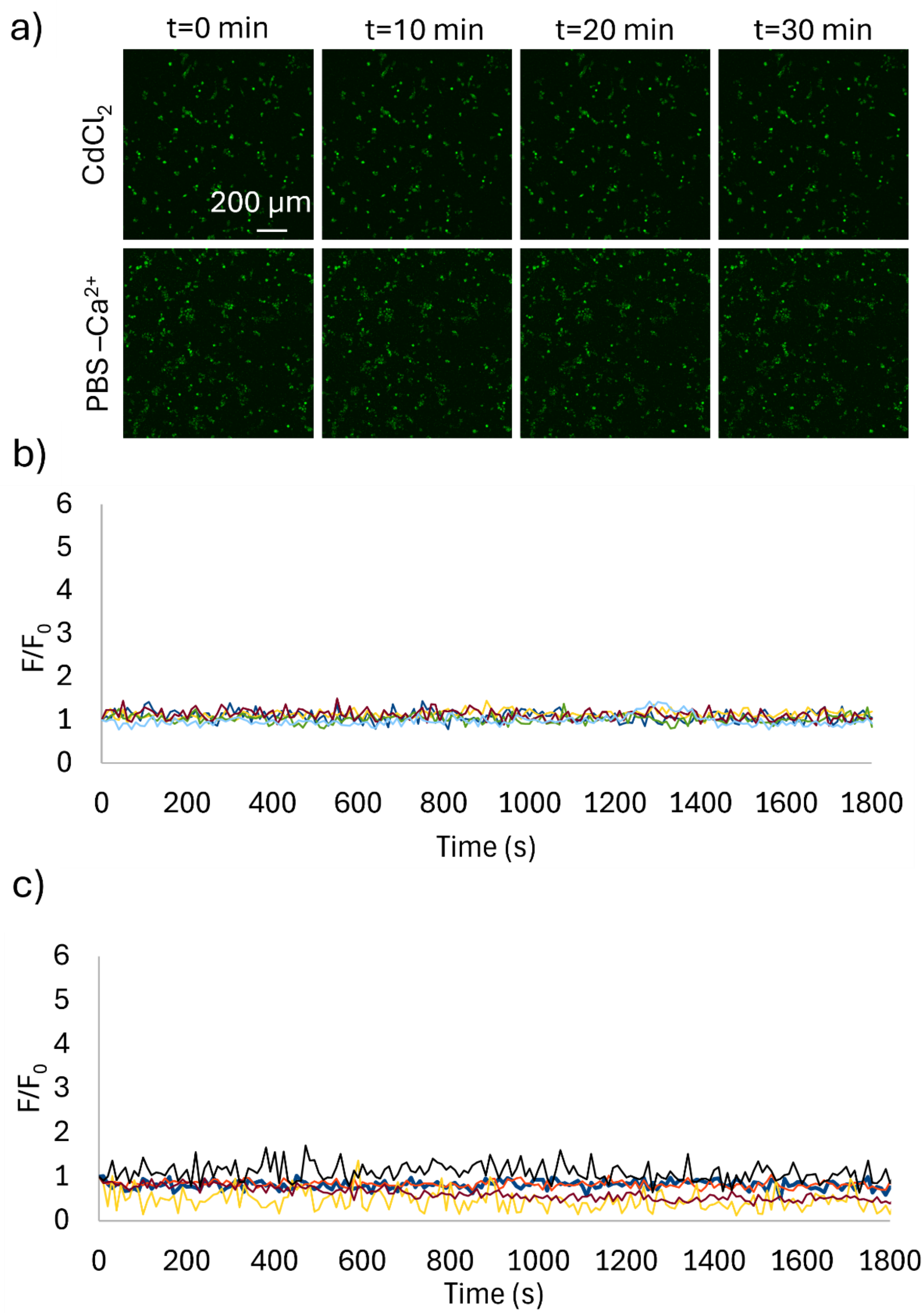

Figure S16. a) Representative confocal fluorescence images of calcium flows in cells treated with PDA-NTs during US in the presence of cadmium chloride or of calcium-free PBS). b) Corresponding quantitative analysis.

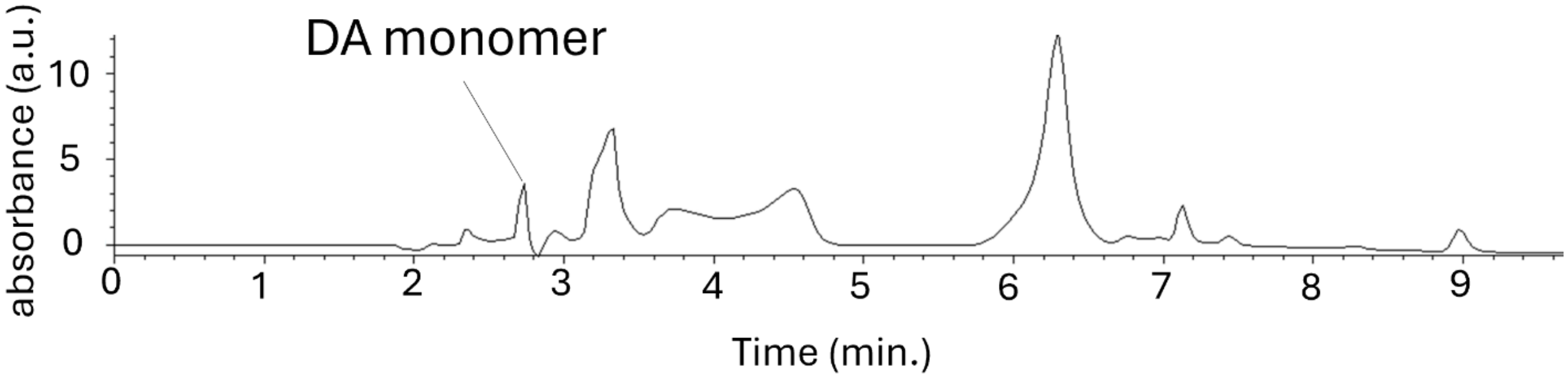

Figure S17. HPLC chromatogram of dopamine-related species released by US-stimulated PDA-NTs (24 h time point).

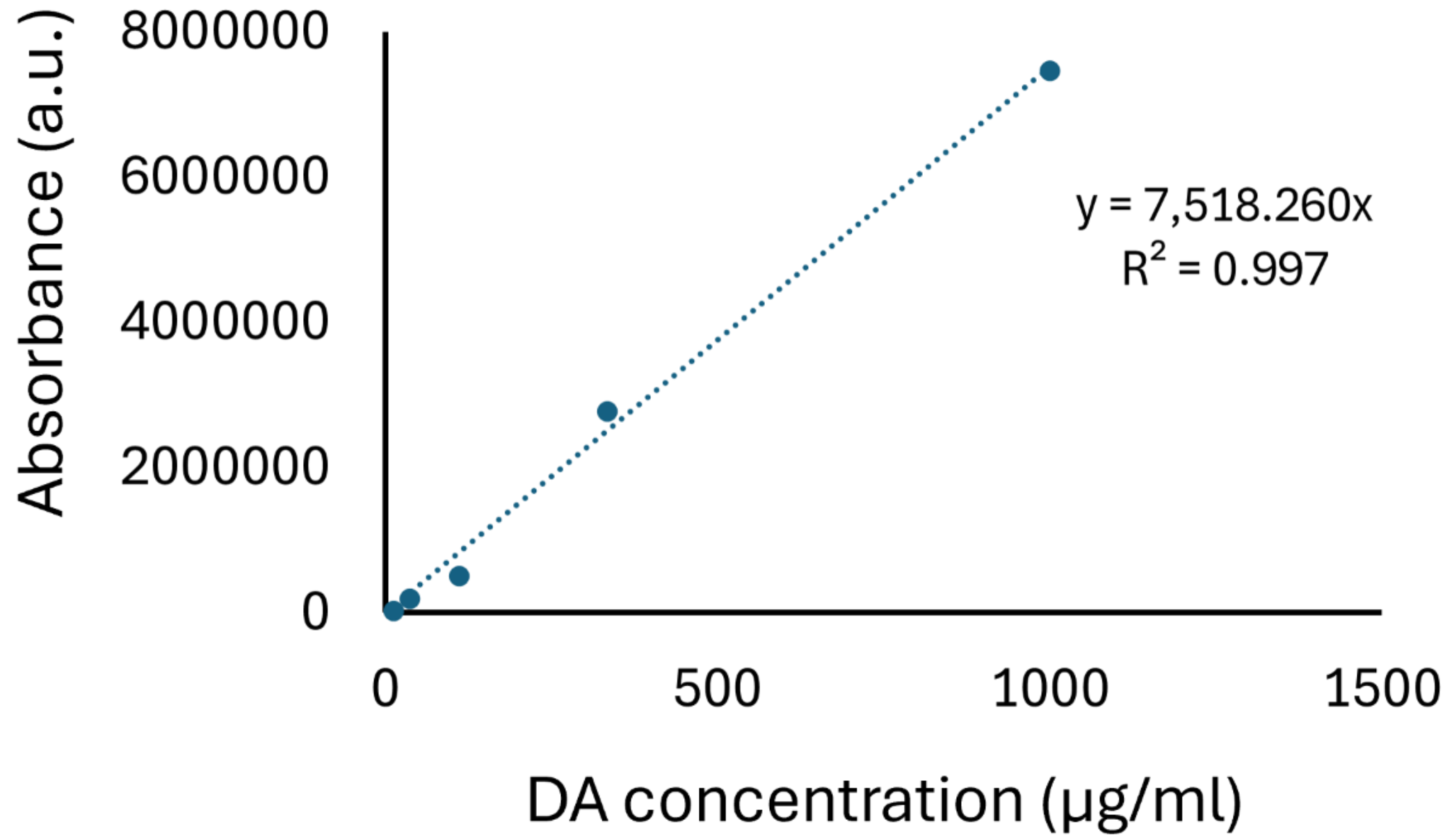

Figure S18. Calibration curve for DA concentration assessment through HPLC.

Video S1. Calcium imaging of U87 MG cells during ultrasound (US) stimulation in the absence of PDA-NTs (control condition). Cells showed minimal spontaneous calcium activity and no significant synchronized calcium response upon US stimulation.

Video S2. Calcium imaging of U87 MG cells during ultrasound (US) stimulation in the presence of PDA-NTs. Transient intracellular calcium spikes and characteristic “on/off” calcium oscillations were observed upon US stimulation, consistent with PDA-NT-mediated piezoelectric activation.
